# High-Strength, Self-Sensing Multiphase Hydrogels for Load-Bearing Actuation and Logical Human–Machine Interaction

**DOI:** 10.1007/s40820-026-02280-y

**Published:** 2026-07-23

**Authors:** Zhilin Zhang, Jiayi Gu, Lina Wang, Xingchen Cui, Xu Zhai, Yue Xu, He Liu, Deliang Li, Bingle Li, Ye Tian, Baoyang Lu, Yu Fu, Tieqiang Wang

**Affiliations:** 1 Department of Chemistry, College of Sciences, Northeastern University, Shenyang, 110819 People’s Republic of China; 2 College of Medicine and Biological Information Engineering, Northeastern University, Shenyang, 110169 People’s Republic of China; 3 Jiangxi Province Key Laboratory of Flexible Electronics, Jiangxi Science and Technology Normal University, Nanchang, 330013 People’s Republic of China; 4 School of Chemical and Environmental Engineering, Sichuan University of Science and Engineering, Zigong, 643000 People’s Republic of China

**Keywords:** Multicontinuous-interpenetrating network, High-strength, Actuator, Information encoding system, Human–machine interaction

## Abstract

Stimuli-responsive shape-changing hydrogels are the most competitive candidates for artificial muscles, electronic skins, and soft robotics. However, existing actuating hydrogels often suffer a trade-off between actuation performance and mechanical strength, which greatly limits their application prospects as actuators under external force loads. Here, we adopt a cascade polymerization strategy to successively introduce electrical sensing and mechanically enhanced polymer network phases into sponge-like PNIPAM hydrogels to achieve PNIPAM-based photothermal-responsive actuating hydrogels with fast response, high strength, and self-sensing performance. The as-prepared hydrogel actuator can execute rapid actuation missions even under external loading far exceeding its own mass and generate differentiated electrical sensing signals according to the magnitude of the external load. Based on the corresponding relationship between the mass of the load and the actuation behavior (such as "0/1" encoding), we develop a novel material-based binary information encoding system. Furthermore, by manufacturing logic gates to analyze differentiated feedback sensing signals and integrating them with Internet of Things technology, a closed-loop logic control system is established for remote logic-based interactive communication. This study fills the gap of traditional hydrogels in load-bearing actuation and complex interactive applications and opens up a new direction for the next generation of smart soft materials.

**Supplementary Information:**

The online version contains supplementary material available at 10.1007/s40820-026-02280-y.

## Highlights

- The cascade polymerization synergistic toughening mechanism overcomes the problem of mutual constraints between "mechanical strength and response speed" of traditional hydrogels and realizes load-bearing actuation.
- The “mass–deformation” dynamic coding law was established, enabling a hydrogel-based 0/1 binary information encoding system derived from mechanical actuation responses.
- Actuation–perception integrated logic-gate units were developed to enable intelligent soft robotics through a closed-loop “light-triggered actuation–signal feedback–logic processing–robotic action” control system.

**Supplementary Information:**

The online version contains supplementary material available at 10.1007/s40820-026-02280-y.

## Graphical abstract

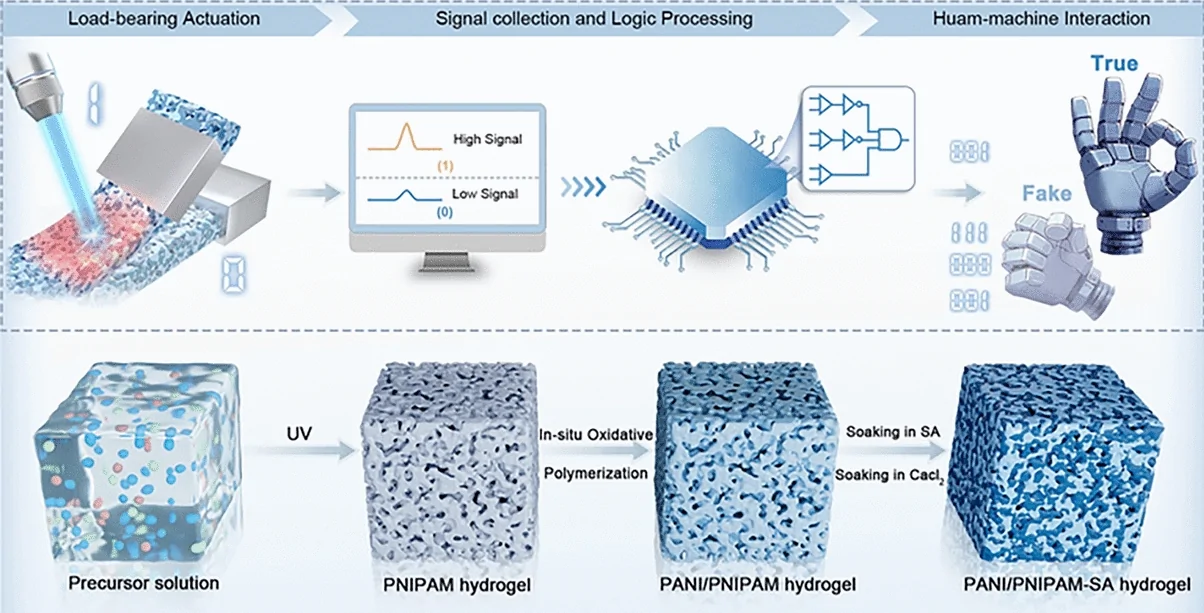

## Introduction

Shape-transformation soft actuators that can undergo reversible volume or shape changes in response to external stimuli (such as temperature, light, magnetic field, pH, or ionic strength [1–6]) have attracted broad interest in artificial muscles and biomimetic soft robots [7, 8]. Among all soft actuating materials, stimuli-responsive hydrogels are highly promising candidate materials for soft robotics due to their resemblance to biological tissues [9–11]. However, the intrinsic mechanical strength of traditional stimuli-responsive hydrogels is insufficient, rendering them susceptible to structural failure under external loads [12–14]. Meanwhile, the energy generated by volume contraction is often insufficient to overcome gravitational forces, particularly when lifting objects exceeding the hydrogel’s own mass [15, 16]. These limitations seriously restrict the actuation performance and application scenarios of stimuli-responsive hydrogel-based soft actuators, especially under external loads. The aforementioned limitations stem from two fundamental issues: (1) the lack of mechanical robustness in single-network hydrogels, which restricts stress transmission [17] and (2) the absence of appropriate structural design to disperse stress resulting in failure under load [18, 19]. Therefore, developing hydrogel actuators with adequate mechanical strength is highly desirable especially aiming to load-bearing actuation tasks under complicated external force environment.

In recent years, although the modulus of responsive hydrogel has been significantly improved through introducing rigid mechanical reinforcement phases (such as nano-clays and carbon nanotubes) within the hydrogel matrix [20, 21], the densified chain structure will also trigger a dramatic reduction in response rate by 1–2 orders of magnitude [22, 23]. The essence of this “high mechanical strength-low response rate” contradiction stems from the non-synergistic effect of the reinforcement phase and the polymer network: While the reinforcement phase enhances the mechanical strength by increasing intermolecular interaction and densifying the chain structure, two essential factors for the volume phase change of the responsive hydrogel (dynamic rearrangement ability of the polymer chain segments and transport of water molecules across hydrogel matrix) are also hindered seriously, thus, resulting in dramatic restriction of the actuation performance of the hydrogel actuators [24–27]. Overcoming the trade-off between mechanical reinforcement and actuation performance to establish hydrogel actuation systems possessing both high load-bearing capacity and rapid actuation response has emerged as a critical scientific challenge, especially for actuating applications subjected to heavy external loads. Meanwhile, it is also ideally anticipated to integrate in-time self-sensing capabilities within the hydrogel actuation system. In that case, the feedback signals during the actuation procedure would be extremely beneficial for logical recognition and judgment against different external force environment, which is of great significance for closed-loop control in advanced robotics, such as industrial grippers and human–machine interactive systems [28–33].

**Fig. 1:**
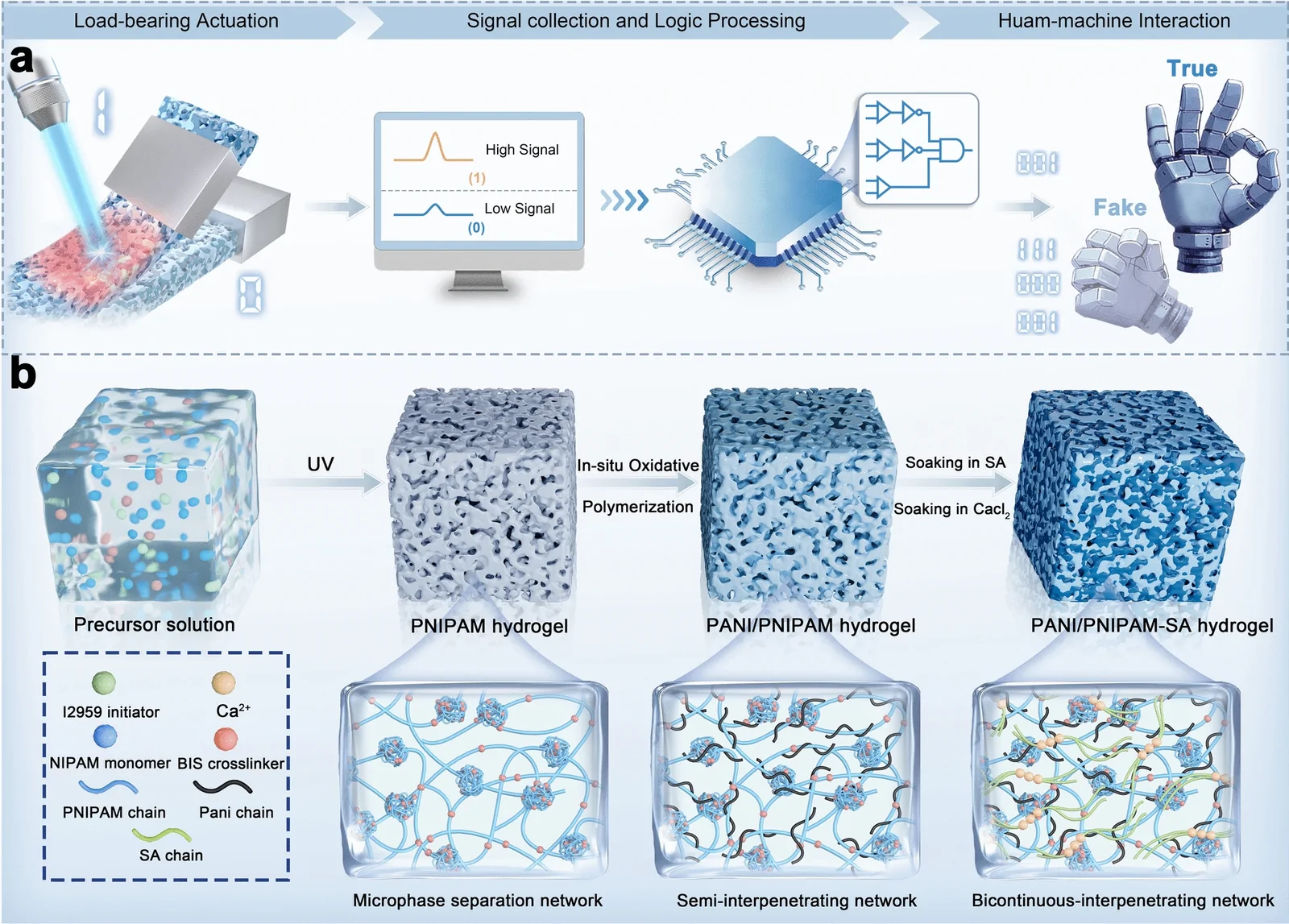
Schematic diagram of the logical human–computer interaction application and fabrication procedure of the continuous-interpenetrating high-strength self-sensing hydrogel. **a** Human–computer interaction through logical analysis of hydrogel’s actuation behavior and feedback sensing signals under load state. **b** The PANI-PNIPAM/SA hydrogel actuator is prepared through a sequential cascade polymerization strategy combining microphase separation polymerization of PNIPAM, in situ oxidative polymerization of PANI, and Ca^2+^ induced crosslinking of SA

To achieve hydrogel actuators featuring load-bearing actuation, rapid stimuli-response, as well as intelligent self-sensing capability through the synergistic effect of network structure, we herein propose a cascade polymerization strategy to in situ oxidatively polymerize aniline (ANI) monomers as the electrical phase within sponge-like poly (N-isopropylacrylamide) (PNIPAM) hydrogels, which are then immersed in a sodium alginate (SA) solution to introduce short polymer chains as the mechanical reinforcement phase, forming a multi-scale synergistic network structure and fabricating a mechanically tunable high-strength self-sensing hydrogel actuator. Benefiting from the highly interconnected spongy porous structure and the introduction of SA mechanical reinforcement phase, the as-prepared PANI-PNIPAM/SA (PPS) hydrogel not only shows rapid stimuli-response, but also features further improved mechanical strength, which enables the PPS actuator to achieve rapid actuation deformation even under loading of external force. Meanwhile, the continuous conductive pathways formed by the embedded PANI network also endows the PPS hydrogel with remarkable sensing capabilities to generate distinguishable electrical feedback sensing signals according to actuation behavior under different external force. Together with all these synergistic characteristics, a novel material-based binary information encoding system was established based on the actuation behavior of the PPS actuators under different external loads (“0” for contraction and “1” for inflection). Moreover, by logically analyzing the distinguishable feedback sensing signals and integrating with Internet of Things (IoT) technology, logical interactive communication between soft and rigid robotic systems was further constructed, enabling binary information transmission through human–machine collaboration (Fig. 1a). Notably, this study achieves synergistic integration of high strength, rapid response, and intelligent perception through material design and functional convergence, addressing critical limitations of conventional actuating hydrogels in load-bearing actuation and complex interactive applications. This work not only provides fundamental insights into designing high-performance hydrogel actuators but also opens new avenues for intelligent soft materials in next-generation human–machine interfaces and logic-based interactive systems.

## Experimental Section

### Materials

N-isopropylacrylamide (NIPAM) and 2-Hydroxy-4'-(2-hydroxyetho-xy)-2-methylpro-piophenone (initiator, 2959) were purchased from TCI (Shanghai) Development Co., Ltd. N, N'-methylenebisacrylamide (BIS), Aniline (ANI), and Calcium chloride (CaCl_2_) were purchased from Energy Chemical (Shanghai). Ammonium peroxodisulfate (APS) was purchased from Alfa Aesar (Tianjin) Chemical Co., Ltd. Sodium alginate (SA) was purchased from Aladdin. All the above reagents were used without further treatment.

### Preparation of Sponge-Like PNIPAM, PANI-PNIPAM and PPS Hydrogel

2 g of NIPAM powder was dissolved in 8 mL deionized water, followed by vigorously stirred for 30 min to obtain a 20 wt% NIPAM monomer homogeneous solution. Then, 0.02 g BIS (2 mg mL^−1^) and initiator 2959 (I2959) (2 mg mL^−1^) were added to the NIPAM solution under continuous stirring to obtain PNIPAM hydrogel precursor solution. Subsequently, the aqueous solution was bubbled with N_2_ for 15 min to remove the air in water. The aqueous solution with NIPAM monomer, BIS crosslinker and I2959 photo-initiator was cast into a special silicone mold (70 mm (length) × 70 mm (width) × 1 mm (thickness)) and exposed to UV light (365 nm, 2 W cm^−2^) at temperature above LCST for 30 min to form sponge-like PNIPAM hydrogel (abbreviated as S-PNIPAM). Finally, the sponge-like PNIPAM hydrogel was immersed in deionized water for 24 h to wash out the unreacted monomer, during which the deionized water was exchanged at least three times. For comparison, a control PNIPAM hydrogel sample was also prepared at room temperature (named as normal PNIPAM hydrogel, N-PNIPAM).

The PANI-PNIPAM hydrogels were prepared by in situ oxidative polymerization of aniline within the sponge-like PNIPAM hydrogels. Firstly, the fully swollen PNIPAM hydrogel was freeze-dried to remove the water in the matrix. Then, it was soaked in 10 mL hydrochloric acid solution (0.1 M) with 0.2 M aniline monomer, fully swelled for 6 h to make the aniline monomer evenly distributed in the PNIPAM hydrogel matrix. Subsequently, 10 mL of precooled hydrochloric acid (0.1 M) with 0.2 M APS was added into the above solution in an ice bath and react for 12 h to prepare PANI-PNIPAM hydrogel. Finally, the PANI-PNIPAM hydrogel was washed with deionized water to remove unreacted reagents and free ions.

PANI-PNIPAM/SA hydrogel was prepared by soaking PANI-PNIPAM in sodium alginate solution (0.6 wt%) for 24 h, and then soaking it in calcium chloride solution (1 M) for a certain time to crosslink sodium alginate. Similarly, the performance of PANI-PNIPAM/SA hydrogel could be adjusted by changing the concentration of sodium alginate solution and the immersion time in calcium chloride solution. Unless otherwise specified, samples soaked in 0.6 wt% sodium alginate solution for 24 h and crosslinked within calcium chloride solution for 900 s will be used for subsequent testing.

### Characterization

Scanning electron microscopy (SEM) micrographs were taken by Hitachi SU8010 at an acceleration voltage of 5 kV. Fourier transform infrared (FTIR) spectra were recorded by an IR spectrophotometer (Bruker VERTEX70) in the scanning range of 4000–400 cm^−1^, the samples were obtained by a KBr disk technique. Mechanical performance tests were performed using a universal testing machine (ZQ-990LB, ZHIQU Precision Instrument) at room temperature on hydrogel specimens (25 mm × 2 mm × 1 mm) at a tensile speed of 50 mm min⁻^1^. The LCR electrical signal changes of the PPS hydrogel under tensile deformation and near-infrared stimulation were recorded using an LCR meter (Tonghui, TH2829C). Photothermal bending tests were conducted on hydrogel strips (20 mm × 2 mm × 1 mm) fixed at one end in a container and irradiated at the midpoint with an NIR laser; the underwater deformation was recorded in real time using an iPhone and tuned by varying the NIR intensity. Swelling/deswelling kinetics were evaluated in 50 °C and 25 °C water baths by periodically removing the hydrogel, blotting the surface, and recording its mass. Water retention was calculated as *WR* = (*W*_*t*_ − *W*_*d*_)/(*W*_*0*_ − *W*_*d*_) × 100%, where *W*_*t*_ is the mass at time t (at 50 °C or 25 °C), *W*_*0*_ is the mass at full swelling, and *W*_*d*_ is the dry mass. To quantitatively characterize the displacement evolution and bending dynamics under external load constraints, vertical lifting and eccentric-load bending tests were performed using hydrogel samples with geometrical shape of 70 mm × 15 mm × 1 mm and 20 mm × 2 mm × 1 mm, respectively.

## Results and Discussion

### Preparation of the PANI-PNIPAM/SA (PPS) Actuating Hydrogel

The PANI-PNIPAM/SA (PPS) actuating hydrogel was prepared through a sequential cascade polymerization strategy including microphase separation polymerization of PNIPAM, in situ oxidative polymerization of PANI, and Ca^2+^-induced crosslinking of SA (Fig. 1b). The thermoresponsive PNIPAM hydrogel matrix with highly interconnected spongy porous structure was initially synthesized via temperature-induced microphase separation polymerization [34–36]. During polymerization at elevated temperatures (T > LCST), polymerized PNIPAM chains undergo hydrophobic collapse, driving the formation of dense polymer clusters within the loose network. This phase separation mechanism creates interconnected, sponge-like micropores, which facilitate efficient water migration across the hydrogel matrix, playing an important role for rapid actuation response. Notably, while the high crosslinking density enhances mechanical robustness, the denser network structure greatly influences its thermoresponsive deswelling behavior at temperatures above LCST (Fig. S1). Subsequently, PANI was in situ polymerized within the sponge-like PNIPAM matrix, where its conductive chains anchored to hydrophobic domains via π–π stacking, mimicking the neural network in muscle tissue to achieve self-sensing feature based on the chain migration during actuation deformation. Meanwhile, the highly efficient light-to-heat conversion of PANI also endows the hydrogel actuator with rapid photothermal-responsiveness. After the introduction of conductive PANI, the composite hydrogel was then infiltrated with short-chain SA based on hydrogen-bond interactions with both PNIPAM and PANI to predefine crosslinking sites. Finally, selective Ca^2+^ coordination with SA carboxylate groups formed a dynamic “egg-box” ionic network, which synergistically reinforced mechanical integrity with little influence on responsiveness (Fig. S2). Fourier transform infrared (FTIR) spectra of the hydrogel matrix at different synthesis stages further indicate the successful introduction of PANI and Ca^2+^-crosslinked SA components within the PSS hydrogel matrix after the sequential cascade polymerization (Fig. S3). Notably, the Ca signal in the XPS survey spectrum and the characteristic Ca^2+^ peaks in the high-resolution Ca 2p spectrum support the formation of Ca^2+^-mediated ionic coordination/crosslinking in the PSS network (Fig. S4). Moreover, SEM–EDS elemental mapping (Fig. S5) reveals that both Cl and Ca are homogeneously distributed throughout the hydrogel framework. Considering that Cl originates from the HCl-doped PANI phase and Ca from the SA/Ca^2+^-crosslinked phase, these results indicate that the different functional components are integrated across the entire porous network, supporting the formation of a continuous-interpenetrating multiphase structure in the PPS hydrogel.

### Characterization of the PPS Actuating Hydrogel

**Fig. 2:**
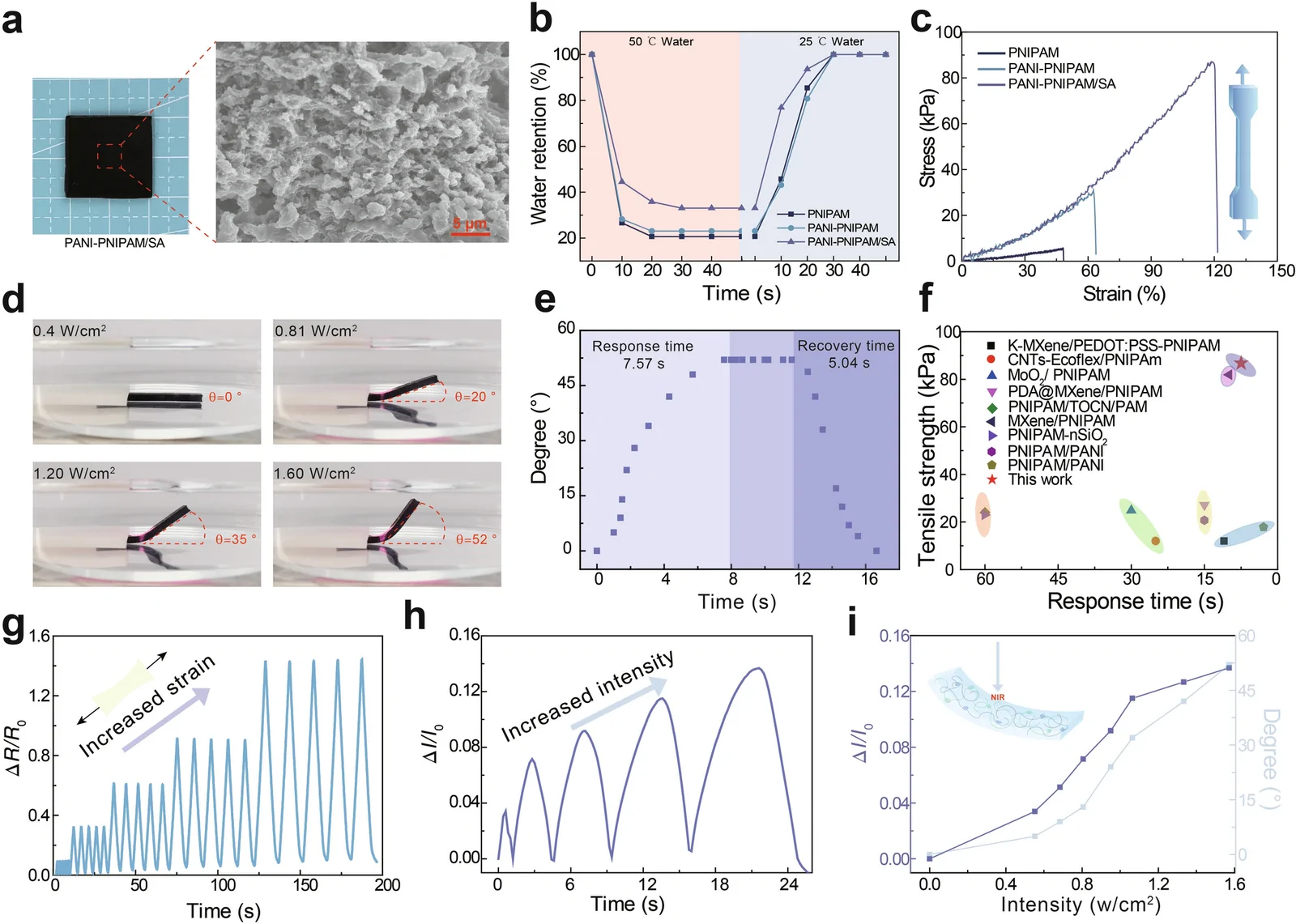
Morphology and basic characteristics of the PPS hydrogel. **a** Optical (left) and SEM images (right) of PPS hydrogel. **b** Deswelling kinetics and Swelling kinetics. **c** Strain – stress curves of hydrogels at different stages of material preparation. i.e., PNIPAM, PANI-PNIPAM (PP) and PANI-PNIPAM/SA (PPS). **d** Optical image of the flectional PPS hydrogel soft actuator under NIR irradiation with different intensities. **e** The real-time bending angle of PPS hydrogel actuator under 1.6 W/cm^2^ NIR irradiation indicates its response rate and recovery rate. **f** Comparison chart by plotting the tensile strength and response time of homogeneous PNIPAM-based hydrogel actuators. **g** Sensing signal of PPS hydrogel stretched with strains ranging from 10 to 100% (10%, 30%, 50%, 70%, and 100% strain, respectively). **h** Real-time electrical sensing signal of PPS hydrogel actuator during intermittently switching on/off the NIR laser with different intensities. **i** Bending angle and electrical signal of PPS hydrogel actuator

As illustrated in the optical photograph and SEM images of the PPS hydrogel (Fig. 2a), the hydrogel shows a black appearance owing to the existence of PANI, and there remain abundant spongy open-celled pores within the hydrogel matrix, even after the introduction of PANI and SA, enabling rapid thermoresponsiveness deswelling and reswelling at different temperature (Fig. S6). The deswelling and swelling kinetics behaviors of S-PNIPAM, PANI-PNIPAM (PP), and PPS hydrogels were compared as shown in Fig. 2b. All the hydrogels reached deswelling equilibrium in ~ 20 s with barely slight decrease of deswelling ratio (from ~ 80% to ~ 70%) upon transferring hydrogels from 25 to 50 °C, and reabsorbed water rapidly to achieve swelling equilibrium in ~ 30 s when decreasing the temperature to 25 °C. It is worthy to note that the rapid response rate is mainly attributed to the sponge-like open-pore structure within the spongy hydrogel matrix. Despite N^2^ adsorption–desorption measurements (Fig. S7) revealed that the accessible porosity gradually decreased from PNIPAM to PP and then to PPS, indicating progressive pore contraction after PANI polymerization and subsequent SA/Ca^2+^ crosslinking, which also induced a corresponding decrease in the equilibrium swelling ratio of the hydrogels (Fig. S8). Compared with the normal PNIPAM hydrogel (N-PNIPAM) with a relatively closed-cell structure, the as-prepared PPS hydrogel still maintain an open and interconnected porous network similar as that of the sponge-like PNIPAM hydrogel (S-PNIPAM) (Fig. S9), which is more favorable for rapid water transport during the thermal-responsive swelling/deswelling process. As a result, the incorporation of conductive PANI and the secondary Ca^2+^-crosslinked SA network has negligible impact on the response rate. Meanwhile, the Ca^2+^-crosslinked alginate network introduces further reversible and dynamic crosslinking sites, resulting in dramatical improvement on the mechanical property of the PPS hydrogel. As shown in the uniaxial tensile tests of hydrogels at different synthesis stages: pure PNIPAM, PP, and PPS (Figs. 2c and S10), the tensile strength increased from 5.56 kPa (pure PNIPAM) to 30.89 kPa (PP) and further to 86.83 kPa (PPS), while Young’s modulus rose from 4.85 kPa (pure PNIPAM) to 23.82 kPa (PP) and 27.61 kPa (PPS). Rheological tests revealed that all hydrogels exhibited typical viscoelastic behavior, with G′′ consistently higher than G′. Relative to PNIPAM, the PP hydrogel displayed enhanced moduli owing to the reinforcing effect of in situ polymerized polyaniline, while the incorporation of sodium alginate together with Ca^2+^-induced ionic crosslinking further strengthened the PPS network (Fig. S10). The PPS hydrogel exhibited greatly improved flexibility and high strength, enduring diverse mechanical deformations—including folding, rolling, stretching, and load-bearing (exceeding 2500 times its own weight, Fig. S12). In contrast, the pure PNIPAM hydrogels failed under high mechanical loads due to inferior strength. This enhancement is mainly attributed to three multi-scale synergy effect: (1) Dual-network synergy: The covalent PNIPAM network constrains large deformations, while the SA-Ca^2^^+^ dynamic network dissipates energy via reversible “egg-box” bond (-COO⁻/Ca^2^^+^) rupture-reformation, mitigating stress concentration. (2) Microphase interface reinforcement: Hydrogen bonding (e.g., -NH of PNIPAM with -OH/-COOH of SA) at the hydrophobic (PNIPAM)-hydrophilic (SA) microphase interface absorbs energy through dynamic bond dissociation under stress, while SA’s ionic crosslinking maintains network stability. (3) Multiscale toughening: SA short chains create localized Ca^2^^+^-crosslinked zones within the PNIPAM network, restricting chain slippage via physical entanglement and ionic interactions, thereby reducing irreversible deformation. Crucially, the material stiffness-toughness balance of the PPS composite hydrogel shows significant relationship with the Ca^2^^+^-crosslinking density, which can be precisely controlled by immersion duration in CaCl_2_ solution (Fig. S13).

Since PANI component not only imparts electrical conductivity but also plays a pivotal role in photothermal conversion, the prepared PPS hydrogel also exhibits excellent photothermal-responsiveness. In contrast to pure PNIPAM hydrogel, owing to the efficient NIR absorption of PANI, the surface temperature of PP and PPS hydrogels reaches PNIPAM’s phase transition temperature rapidly within only 10 s under 1.6 W cm^−2^ NIR irradiation (Figs. S14 and 15), triggering volume contraction (Figure S16). Meanwhile, the surface temperature of PPS hydrogels could be precisely and reversibly tuned by modulating the NIR intensity (Fig. S17). Leveraging the exceptional photothermal conversion capability, the PPS hydrogel also exhibits tunable photothermal-responsive actuation behaviors. A rectangular hydrogel strip (20 mm (length) × 1 mm (width) × 1 mm (thickness)) was anchored at one end to the substrate as a flectional soft actuator. Upon localized near-infrared (NIR) laser irradiation, the asymmetric temperature gradient across the hydrogel induced by localized irradiation will result in anisotropic contraction of the hydrogel, thus further inducing phototropic bending (Movie S1). Crucially, since the local temperature is highly dependent on the irradiation intensity of the NIR laser, the deformation magnitude can be precisely modulated by adjusting the NIR laser intensity (Fig. 2d). At NIR power densities below 0.4 W cm^−2^, insufficient heat generation prevents the PNIPAM network from reaching its phase transition temperature, resulting in negligible bending. Increasing the intensity beyond 0.8 W cm^−2^ elevates the localized temperature above the LCST, driving contraction-dependent bending. By tuning the NIR power density between 0.4 and 1.6 W cm^−2^, the bending angle could be controllably adjusted within 0°–52°, and gravitational potential energy analysis (classical mechanical formulation) revealed a maximum energy gain of 0.588 μJ (Fig. S18). Furthermore, the PPS actuator exhibited fast photothermal response, achieving maximum bending in 7.57 s under NIR irradiation (1.6 W cm^−2^) and recovering to its original state within 5.04 s post-irradiation (Fig. 2e) with excellent cyclic stability over 50 on/off cycles (Fig. S19). This rapid kinetics is attributed to the sponge-like porous framework enabling efficient water migration as well as the high photothermal efficiency of the interpenetrating PANI network. These results demonstrate that the PPS hydrogel synergizes exceptional mechanical properties with rapid responsivity, outperforming conventional homogeneous PNIPAM-based hydrogels (Fig. 2f and Table S1).

Beyond actuation, the PANI chain migration during shape deformation also endows the PPS hydrogel with dual strain- and actuation-sensing capabilities. Uniaxial stretching tests reveal an obvious strain-dependent resistance changes with considerable sensitivity (Gauge Factor, GF = 1.36) and linearity (R^2^ = 0.99) across 0–100% strain (Figs. 2g and S20). What’s more, real-time electrical signal (△I/I_0_) monitoring during intermittently increasing NIR irradiation intensity further demonstrates that the feedback electrical signal is highly related with the NIR-responsive actuation of the PPS hydrogel actuator (Fig. 2h). Notably, the plot data of the feedback electrical signal and the balanced deformation extent (folding angle) during actuation under different NIR irradiation intensity (Fig. 2i) clearly demonstrate that the feedback electrical signal is also positively correlated with the intensity of NIR laser, as well as the folding angle of the soft actuator. In other words, through monitoring the feedback electrical signal, it is possible to realize self-sensing of the real-time deformation extent during the actuation process of the PPS actuator. Moreover, the multiphase interpenetrated structure also endows the soft actuator with excellent sensing stability in both 200-cycle uniaxial stretching test and 200-cycle NIR-induced actuation (Figure S21 and S22), underscoring its reliability for soft robotics.

### Load-Bearing Actuation Performance of the PPS Hydrogel Actuator

**Fig. 3:**
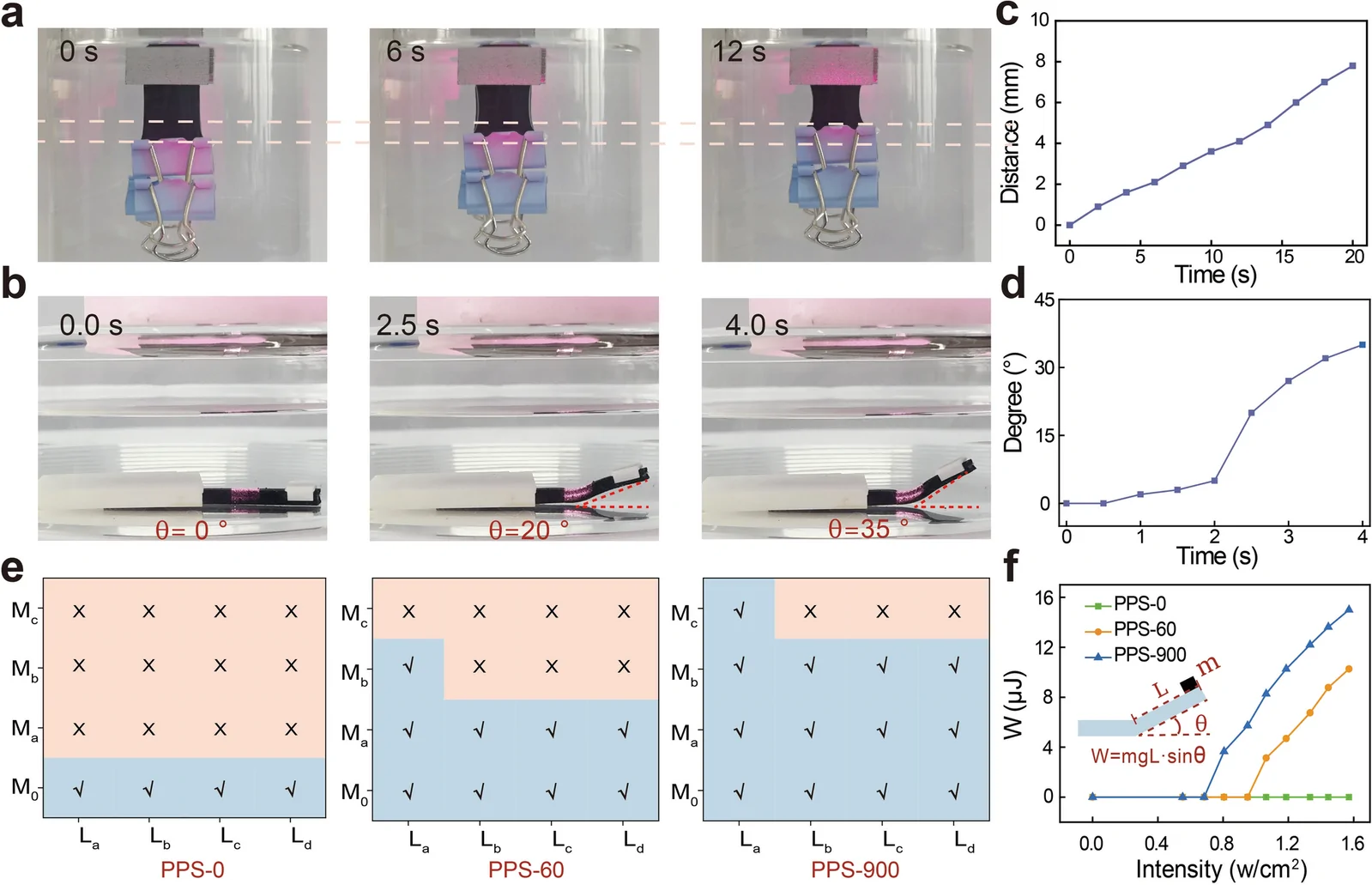
Load-bearing photothermal actuation and energy output of PPS hydrogel actuators. **a** Photographs showing the PPS actuator lifting a suspended object (300 × its dry weight) under NIR irradiation (0, 6, and 12 s). **b** Photographs of NIR-triggered bending PPS actuator under eccentric-load configuration (0, 2.5, and 4.0 s). **c** Corresponding lifting displacement (distance) as a function of time during the load-bearing lifting actuation. **d** Corresponding bending angle versus time during the bending actuation under eccentric-load configuration. **e** Actuation state phase diagrams constructed by systematically varying the payload mass *m* (m_a_ = 0.3 g, m_b_ = 0.6 g, m_c_ = 1.0 g) and lever arm *L* (relative to the hydrogel center, L_a_ = 1.5 mm, L_b_ = 2.5 mm, L_c_ = 3.5 mm, L_d_ = 5 mm), with the external torque defined as *τ* = *mgL* (√, actuated; × , non-actuated). **f** Mechanical energy output W of PPS-0, PPS-60, and PPS-900 under an identical loading configuration (fixed *m* and *L*) as a function of NIR power density; *W* was calculated from the experimentally measured lifting height (*Δh*) based on the equation of *W* = *mgΔh*

Given that the composite PPS hydrogel exhibits markedly enhanced mechanical performance compared with pure PNIPAM hydrogel, we further evaluated its load-bearing actuation performance under two representative loading conditions. Specifically, vertical lifting and eccentric-load bending were employed to quantitatively characterize the displacement evolution and bending dynamics under external load constraints (Fig. 3a, b). Under NIR irradiation (1.6 W cm^−2^), the soft robot can lift object 300 × its dry weight, with the lifting height increasing monotonically and reaching ~ 8 mm within ~ 20 s (Fig. 3c and Movie S2). When the weight was applied in an eccentric-load configuration, the hydrogel likewise maintained rapid bending output, with the bending angle increasing from 0° to ~ 35° within ~ 4 s and quickly approaching a steady state (Fig. 3d), indicating that effective deformation and mechanical output are retained under the coupled constraints of gravity and external loading.

For the PPS hydrogel actuator, the load-bearing actuation capacity mainly originated from the mechanical reinforcement effect by Ca^2+^-crosslinked alginate network. Therefore, through adjusting the immersion duration in CaCl_2_ solution (1 M) to regulate the crosslinking density of the alginate network, it is possible to adjust the load-bearing photothermal actuation capacity of the PPS hydrogel actuator. To achieve this, three different hydrogel variants—PPS-0 (un-crosslinked SA), PPS-60 (partially crosslinked SA), and PPS-900 (fully crosslinked SA)—were fabricated through crosslinking the PPS hydrogel in CaCl₂ solution for different immersion duration (0, 60, and 900 s, respectively). With increasing immersion time, the crosslinking density and mechanical strength of the PPS hydrogels progressively increased. Subsequently, we adopted the torque, *τ* = *mgL*, as a unified descriptor to systematically vary the payload mass (*m*) (Fig. S23) and its loading distance (*L*, lever arm) relative to the hydrogel center, thereby constructing an “actuated/non-actuated” state phase diagram to determine whether an appreciable bending response could be triggered under different (*m*, *L*) combinations (Fig. 3e). This phase map directly delineates the actuation boundary under gravity-opposed loading and shows that, with prolonged crosslinking, the PPS hydrogels can sustain triggerable bending over a broader range of higher torques, with PPS-900 exhibiting the widest actuating domain. Furthermore, to compare the energy-delivery capability of hydrogels with different mechanical strengths under load constraints, we fixed the loading configuration (constant *m* and *L*) and varied only the NIR power density, while quantifying the gravitational work using the experimentally measured lifting height *Δh*, i.e., *W* = *mgΔh*. As the irradiation intensity increased, the work output increased for all samples; notably, PPS-900 achieved a larger *Δh* and a markedly higher *W* under the same loading configuration, outperforming PPS-60 and PPS-0 overall, which clearly demonstrates that mechanical reinforcement enhances the effective mechanical output under load (Fig. 3f). Meanwhile, we statistically analyzed the steady-state bending angle (*θ*) of PPS-900 under different applied torques and established an empirical *θ* – *τ* fitting relationship (Fig. S24), providing a quantitative calibration basis for subsequently converting “loading conditions” into discernible bending states for information encryption.

### Binary Information Encoding Based on Load-Bearing Actuation of the PPS Hydrogel Actuator

**Fig. 4:**
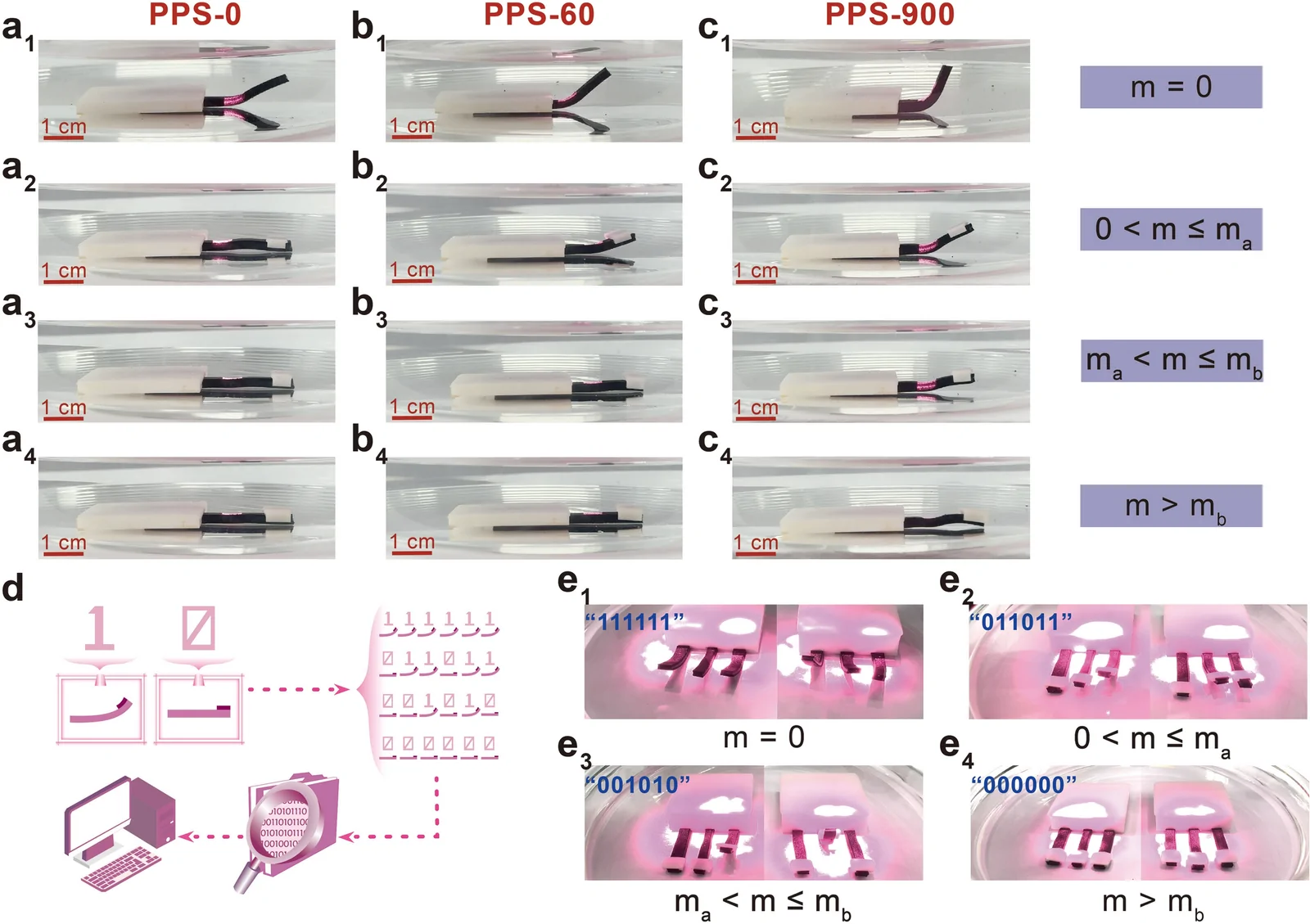
Actuation performance and binary information encoding application of mechanically adjustable PPS hydrogel under load. The actuation behavior under NIR irradiation (808 nm, 1.6 W/cm^2^) for **a**_**1**_**–a**_**4**_ PPS-0 hydrogel, **b**_**1**_**–b**_**4**_ PPS-60 hydrogel, and **c**_**1**_**–c**_**4**_ PPS-900 hydrogel with varying mass loads. **d** Schematic diagram of the binary information encoding principle. **e**_**1**_**–e**_**4**_ Passwords displayed by the PPS hydrogel array based on its actuation behavior under varying mass loads

Since the PPS hydrogels with different crosslinking densities in the alginate network exhibit distinguishable load-bearing actuation capacities, it is possible to construct a novel material-based binary information encoding system by integrating PPS hydrogel actuators with different load-bearing actuation capacities. As shown in Fig. 4a-c, three different PPS hydrogel actuators exhibit different photothermal actuation behaviors (Under NIR irradiation, 1.6 W cm^−2^) upon gradually increasing the external load (Movie S3). The PPS-0 actuator, which exhibits inferior mechanical properties, can only execute bending actuation under non-load condition, and lose bending actuation capacity under any applied load owing to insufficient energy to overcome gravitational forces (Fig. 4a_1_–a_4_). The PPS-60 actuator with moderate mechanical properties demonstrates partial load tolerance: Bending occurs at loads ≤ m_a_ (0.3 g) but fails at higher loads (Fig. 4b_1_–b_4_). Remarkably, owing to the reinforced mechanical properties, the PPS-900 actuator can lift masses up to ~ 0.6 g (m_b_, 30 × its dry weight) with obvious bending actuation behavior, featuring robust load-bearing actuation capacity (Fig. 4c_1_–c_4_). Capitalizing on this intriguing phenomenon, we developed a novel material-based binary information encoding system (Fig. 4d and Movie S4) that leverages the load-dependent actuation behavior of the photothermal-responsive PPS hydrogel actuators, encoded as binary states ("0" or "1"), distinct from conventional electronic or optical information processing methods. Under NIR laser irradiation, hydrogels with tailored mechanical properties exhibit differential phototropic bending behaviors depending on applied load mass: a “1” is assigned if bending occurs, whereas “0” denotes the absence of bending. A six-unit PPS hydrogel actuator array was engineered to function as a logic-based binary encoding system (Fig. S25), generating dynamic binary codes under varying load conditions. For instance: Load = 0: Code “111,111”; Load = m_a_ (0.3 g): Code “011011”; Load = m_b_ (0.6 g): Code “001010”; Load = m_c_ (1.0 g): Code “000000” (Fig. 4e_1_–e_4_). Here, the predefined binary pattern “001010” serves as a target encoded output, while the load mass functions as a stimulus parameter, where only a specific load condition (e.g., m_b_) generates the corresponding binary sequence. Furthermore, reconfiguring the array’s spatial arrangement enables the generation of higher complexity binary patterns for advanced information encoding. This approach uniquely integrates mechanical load as a physical encoding variable, providing a material-based strategy for stimulus-dependent binary information generation beyond conventional electronic systems.

### Logical Human–Machine Interaction Based on Self-Sensing Feature of the Load-Bearing PPS Hydrogel Actuator

**Fig. 5:**
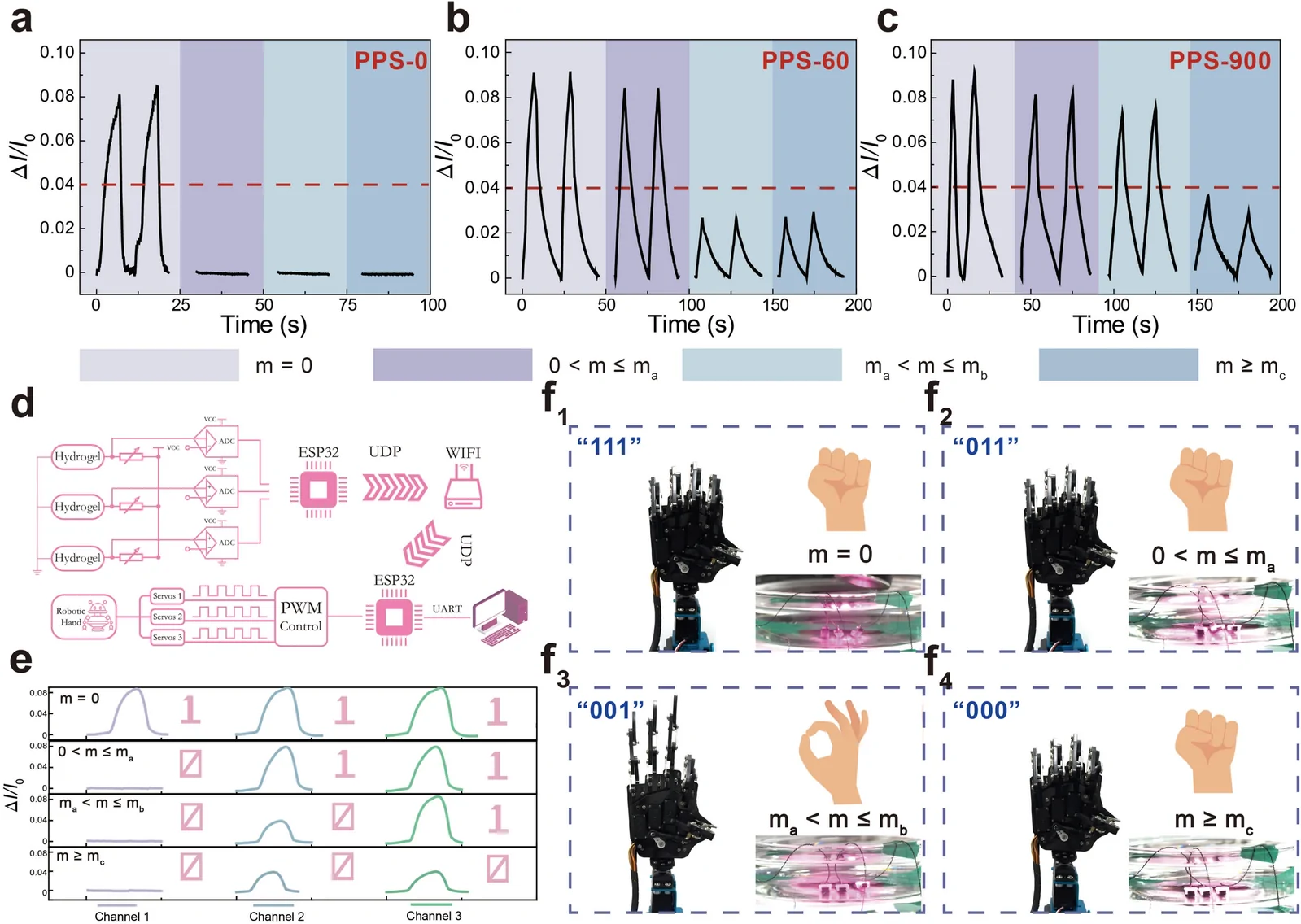
Self-sensing performance and human–computer interaction application of mechanically adjustable PPS hydrogel under load. Real-time electrical signals of the **a** PPS-0, **b** PPS-60 and **c** PPS-900 hydrogel actuator during actuation under varying mass loads. **d** Schematic diagram of the human–machine interaction logic. **e** Real-time electrical signals and the decoded binary sequence of the hydrogel actuator array assembled in the order PPS-0, PPS-60, and PPS-900 when actuating under different external loads. **f**_**1**_**–f**_**4**_ The self-sensing actuator array loaded with different masses exhibit distinct behaviors under NIR stimulation, generating unique binary codes, which are then recognized by the robotic arm to perform corresponding gestures

Since the PPS hydrogel actuators show distinct photothermal-responsive deformation behaviors under different external load, the structural change of PANI network in the PPS hydrogel should also differ during the distinct actuation process. Therefore, it is anticipated that the PPS hydrogel actuators should also generate distinct feedback electrical signals, resulting from the PANI chain migration, which might be utilized to perceive the distinct load-dependent actuation behaviors of the hydrogel actuators. To explore the electrical feedback of the PPS during different load-bearing actuation process, real-time electrical signals (ΔI/I₀) of three PPS hydrogel actuators with different load-bearing actuation capacity are monitored during actuation under varying mass loads (Under NIR irradiation (808 nm, 1.6 W cm^−2^)). For the PPS-0 hydrogel actuator (Fig. 5a), the real-time electrical signal changes significantly only under no-load conditions, while shows neglectable changes under any external load due to the limited actuation energy as well as the restrained chain migration (Fig. S26). The PPS-60 hydrogel actuator bends when the external load is less than load threshold (m_b_, 0.3 g), generating obvious electrical signal changes owing to the effective contraction of the polymer networks. However, when the external load of the PPS-60 actuator exceeds its threshold, owing to the loss of bending actuation capacity, the feedback electrical signal declines dramatically. Although there is still a detectable feedback electrical signal, which might result from the horizontal contraction of the hydrogel, the dramatically declined feedback electrical signal can also be clearly distinguished from that generated by bending actuation (Fig. 5b). Similarly, the PPS-900 actuator also produces differentiable feedback electrical signal according to distinct bending/non-bending actuation behaviors under different external load (Fig. 5c). While thanks to its super strong actuation ability of PPS-900 actuator, the differentiated decline of feedback electrical signal only occurs when exceeding its load threshold (m_c_, 0.6 g). Such results indicate that it is possible to judge the load-dependent actuation behaviors between bending and non-bending according to the magnitude of the feedback electrical signal. As concluded in the feedback signals during load-dependent actuation, for all these three PPS hydrogel actuators, the feedback electrical signal for bending actuation is much higher than that for non-bending actuation, thus, we herein manually define an intermediate threshold (ΔI/I₀ = 0.04) to identify the actuation behavior under external load. The feedback electrical signal (ΔI/I₀) greater than the threshold represents bending actuation mode of the actuator, while feedback electrical signal less than the threshold represents the absence of bending actuation.

Most significantly, by means of the differentiable feedback electrical signals corresponding to actuation modes, a closed-loop control system encompassing “light-driven actuation, signal feedback, binary information processing, and action execution” was established by integrating the load-bearing hydrogel actuator array with a robotic manipulator via Internet of Things (IoT) protocols, enabling remote logical interactive communication between soft and rigid robots. Figure 5d schematically illustrates the hybrid system where soft PPS hydrogel actuator array governs the motion of a rigid robotic manipulator. We connected two ESP32 chips to the same local area network to achieve wireless interaction between feedback electrical signal responses generated by NIR-responsive actuation of PPS hydrogel under varying load conditions and the flexion/extension states of the robotic manipulator. The entire system is divided into 4 main parts: 1) PPS self-sensing hydrogel actuator array. 2) Signal acquisition and processing and wireless transmission system, including A/D conversion, digital filtering, and WIFI transmission module. 3) Signal reception and processing system, consisting of WIFI receiving module and servo drive module. 4) Manipulator motion system. Specifically, the PPS hydrogel is connected in series with a 68 kΩ fixed resistor. Under NIR light stimulation, the PPS hydrogel actuators dehydrate to deform, and produce a change in feedback currents. Then, the ESP32 chip collects the real-time electrical signal on the PPS hydrogel actuator and converts it into a digital signal through the A/D conversion module. Next, the WIFI module uses the UDP protocol to remotely transmit it to the ESP32 chip receiver. By detecting the change trend of the signal (rising, falling, or unchanged), the actuation mode of the PPS hydrogel actuator under varying loads is identified and converted into an output command signal to control the flexion/extension of the robotic hand based on predefined logic rules, enabling remote logical interactive communication between the PPS hydrogel soft actuators and the rigid robotic hand. To further analyze the response characteristics of the PPS hydrogel actuator array under NIR stimulation, the real-time feedback electrical signals were also sent to the host computer through the serial port (UART) for visual analysis. Three hydrogel actuators with different load-bearing actuation capacity—PPS-0 (Channel 1), PPS-60 (Channel 2), and PPS-900 (Channel 3)—were aligned sequentially as the soft actuator array. As shown in Fig. 5e, when the aligned actuator array was subjected to NIR laser stimulation under distinct load masses, different channels generated distinct real-time feedback signals corresponding to the applied loads, which were further converted into different binary codes based on a defined decoding principle (ΔI/I₀ > 0.04 represents “1” for bending actuation, whereas ΔI/I₀ < 0.04 represents “0” for the absence of bending actuation). Finally, the resulting binary codes are analyzed and further converted into output instruction signals by the pulse width modulation (PWM) unit according to predefined logic rules, and the output instruction signals are transmitted to the robotic hand to control its flexion/extension states, enabling remote logical interactive communication between the PPS soft actuators and the rigid robotic hand. To clarify the logical interactive communication feature, a binary code “001” was predefined in the logic control program (Table S2). Only when the feedback binary code matched this predefined code did the robotic hand perform an “OK” gesture, whereas no response was triggered (maintaining a persistent “fist” posture) under other feedback codes. Figures 5f and Movie S5 show the response of the rigid robotic hand when the soft actuator array with different external loads is illuminated by an NIR laser (1.6 W cm^−2^). The robotic hand remains in a “fist” posture when the external load applied to the actuator array falls outside the valid load range (m ≤ m_a_ or m > m_c_), owing to the mismatched binary codes generated from the feedback signals of the three channels. Conversely, an external load within the valid load range (m_a_ < m ≤ m_b_) generates the predefined binary code, thereby triggering the robotic hand to perform an “OK” gesture (Fig. 5f_3_). This closed-loop control system innovatively employs the physical load as a mechanical input parameter for the logical interactive communication between PPS soft actuators and the rigid robotic hand, mimicking biological sensory–feedback loops through the “actuation–code–gesture” mapping. The introduction of this load-dependent input parameter increases the complexity of the logical interaction between the soft actuators and the rigid robotic hand, providing an additional physical constraint for the actuation–signal–gesture mapping and paving new avenues for intelligent soft materials in next-generation human–machine interfaces and adaptive communication systems.

## Conclusions

We demonstrate a cascade polymerization strategy to construct a high-strength, fast response, mechanically tunable, self-sensing PPS hydrogel actuator, which is further developed for load-bearing actuation, binary information processing, and soft–rigid robotic logical interaction applications. Thanks to its markedly improved mechanical performance, the PPS hydrogel exhibits greatly enhanced load-bearing actuation capacity, achieving rapid vertical lifting and eccentric-load bending actuations under loading conditions far exceeding its own mass without structural failure. Critically, the load-bearing actuation capacity, resulting from the crosslinking reinforcement of the mechanical properties, can be precisely modulated by adjusting the CaCl₂ immersion time, and a novel material-based information processing system was established by leveraging the distinguished load-dependent actuation behavior of PPS hydrogel actuators. Meanwhile, the incorporation of polyaniline (PANI) established continuous conductive pathways, endowing the PPS hydrogel with remarkable dual strain- and actuation-sensing capabilities with high sensitivity. Benefiting from the differentiable feedback electrical signals corresponding to the actuation mode (bending/unbending) of the PPS hydrogel actuator, integration with Internet of Things (IoT) technology further enabled the development of a closed-loop logical interactive control system, seamlessly linking NIR laser generators, intelligent self-sensing actuators, and an interactive robotic hand. This system pioneers a bioinspired paradigm where mechanical load acts as a physical control parameter, bridging soft material innovation with intelligent human–machine interaction. In summary, this work not only advances the design of intelligent hydrogel actuators with load-bearing capability, but also paves the way for next-generation human–machine interfaces and autonomous soft systems.

## Supplementary Information

Below is the link to the electronic supplementary material.

Supplementary file 1 (MP4 1413 KB)

Supplementary file 2 (MP4 1534 KB)

Supplementary file 3 (MP4 5857 KB)

Supplementary file 4 (MP4 5030 KB)

Supplementary file 5 (MP4 2484 KB)

Supplementary file 6 (DOCX 28142 KB)

## References

[1] C. Lu, W. Chen, X. Zhang, Highly efficient ionic actuators enabled by sliding ring molecule actuation. Nat. Commun. **16**(1), 2480 (2025). 10.1038/s41467-025-57893-5 40074761 10.1038/s41467-025-57893-5 PMC11903884

[2] X. Li, Y. Du, X. Pan, C. Xiao, X. Ding et al., Leaf vein-inspired programmable superstructure liquid metal photothermal actuator for soft robots. Adv. Mater. **37**(18), e2416991 (2025). 10.1002/adma.202416991 39955736 10.1002/adma.202416991

[3] K.-H. Ha, J. Yoo, S. Li, Y. Mao, S. Xu et al., Full freedom-of-motion actuators as advanced haptic interfaces. Science **387**(6741), 1383–1390 (2025). 10.1126/science.adt2481 40146816 10.1126/science.adt2481

[4] C. Xue, Y. Zhao, Y. Liao, H. Zhang, Bioinspired super-robust conductive hydrogels for machine learning-assisted tactile perception system. Adv. Mater. **37**(10), e2416275 (2025). 10.1002/adma.202416275 39901430 10.1002/adma.202416275

[5] Z. Zhang, G. Chen, Y. Xue, Q. Duan, X. Liang et al., Fatigue-resistant conducting polymer hydrogels as strain sensor for underwater robotics. Adv. Funct. Mater. **33**(42), 2305705 (2023). 10.1002/adfm.202305705

[6] F. Gao, H. Jiang, D. Wang, S. Wang, W. Song, Bio-inspired magnetic-responsive supramolecular-covalent semi-convertible hydrogel. Adv. Mater. **36**(29), e2401645 (2024). 10.1002/adma.202401645 38754860 10.1002/adma.202401645

[7] Z. Shen, Z. Zhang, N. Zhang, J. Li, P. Zhou et al., High-stretchability, ultralow-hysteresis conductingpolymer hydrogel strain sensors for soft machines. Adv. Mater. **34**(32), e2203650 (2022). 10.1002/adma.202203650 35726439 10.1002/adma.202203650

[8] W. Yu, W. Zhao, X. Zhu, M. Li, X. Yi et al., Laser-printed all-carbon responsive material and soft robot. Adv. Mater. **36**(36), e2401920 (2024). 10.1002/adma.202401920 39011802 10.1002/adma.202401920

[9] M.C. Koetting, J.T. Peters, S.D. Steichen, N.A. Peppas, Stimulus-responsive hydrogels: theory, modern advances, and applications. Mater. Sci. Eng. **93**, 1–49 (2015). 10.1016/j.mser.2015.04.001 10.1016/j.mser.2015.04.001 PMC4847551 27134415

[10] R. Tognato, A.R. Armiento, V. Bonfrate, R. Levato, J. Malda et al., A stimuli-responsive nanocomposite for 3d anisotropic cell-guidance and magnetic soft robotics. Adv. Funct. Mater. **29**(9), 1804647 (2018). 10.1002/adfm.201804647

[11] X. Liu, M. Gao, J. Chen, S. Guo, W. Zhu et al., Recent advances in stimuli-responsive shape-morphing hydrogels. Adv. Funct. Mater. **32**(39), 2203323 (2022). 10.1002/adfm.202203323

[12] W.J. Li, Q.W. Guan, M. Li, E. Saiz, X. Hou, Nature-inspired strategies for the synthesis of hydrogel actuators and their applications. Prog. Polym. Sci. **140**, 101665 (2023). 10.1016/j.progpolymsci.2023.101665

[13] Y. Zhao, C.-Y. Lo, L. Ruan, C.-H. Pi, C. Kim et al., Somatosensory actuator based on stretchable conductive photothermally responsive hydrogel. Sci. Robot. **6**(53), eabd5483 (2021). 10.1126/scirobotics.abd5483 34043561 10.1126/scirobotics.abd5483

[14] Z.W. Guo, X.Y. Lu, X.H. Wang, X. Li, J. Li et al., Engineering of chain rigidity and hydrogen bond cross-linking toward ultra-strong, healable, recyclable, and water-resistant elastomers. Adv. Mater. **35**(21), e2300286 (2023). 10.1002/adma.202300286 36854256 10.1002/adma.202300286

[15] C.-Y. Lo, Y. Zhao, C. Kim, Y. Alsaid, R. Khodambashi et al., Highly stretchable self-sensing actuator based on conductive photothermally-responsive hydrogel. Mater. Today **50**, 35–43 (2021). 10.1016/j.mattod.2021.05.008

[16] H. Liu, H. Chu, H. Yuan, D. Li, W. Deng et al., Bioinspired multifunctional self-sensing actuated gradient hydrogel for soft-hard robot remote interaction. Nano-Micro Lett. **16**(1), 69 (2024). 10.1007/s40820-023-01287-z 10.1007/s40820-023-01287-z PMC10766940 38175419

[17] T. Zhou, H. Yuk, F. Hu, J. Wu, F. Tian et al., 3d printable high-performance conducting polymer hydrogel for all-hydrogel bioelectronic interfaces. Nat. Mater. **22**(7), 895–902 (2023). 10.1038/s41563-023-01569-2 37322141 10.1038/s41563-023-01569-2

[18] X. Li, J.P. Gong, Design principles for strong and tough hydrogels. Nat. Rev. Mater. **9**(6), 380–398 (2024). 10.1038/s41578-024-00672-3

[19] X. Zhao, X. Chen, H. Yuk, S. Lin, X. Liu et al., Soft materials by design: unconventional polymer networks give extreme properties. Chem. Rev. **121**(8), 4309–4372 (2021). 10.1021/acs.chemrev.0c01088 33844906 10.1021/acs.chemrev.0c01088 PMC9217625

[20] Z. Liu, Y. Faraj, X.J. Ju, W. Wang, R. Xie et al., Nanocomposite smart hydrogels with improved responsiveness and mechanical properties: a mini review. J. Polym. Sci. B Polym. Phys. **56**(19), 1306–1313 (2018). 10.1002/polb.24723

[21] P. Tang, H. Yan, L. Chen, Q. Wu, T. Zhao et al., Anisotropic nanocomposite hydrogels with enhanced actuating performance through aligned polymer networks. Sci. China Mater. **63**(5), 832–841 (2020). 10.1007/s40843-019-1236-8

[22] X. Peng, H. Li, J. Xu, C. Lan, J. Liu et al., Reprogrammable shape morphing hydrogel modulated by synergistic photochromism and photoactuation. Chem. Eng. J. **511**, 162103 (2025). 10.1016/j.cej.2025.162103

[23] W. Feng, F. Li, Z. Jiang, C. Yue, G. Yin et al., Supramolecular entanglement driven emissive aggregate densification enabling room-temperature phosphorescence hydrogels with ultrastretchability and crack-tolerance. Angew. Chem. Int. Ed. **64**(29), e202505192 (2025). 10.1002/anie.202505192 10.1002/anie.202505192 40347063

[24] S. Palagi, A.G. Mark, S.Y. Reigh, K. Melde, T. Qiu et al., Structured light enables biomimetic swimming and versatile locomotion of photoresponsive soft microrobots. Nat. Mater. **15**(6), 647–653 (2016). 10.1038/nmat4569 26878315 10.1038/nmat4569

[25] B. Zhang, X. Cui, W. He, L. Shao, T. Wang et al., Asymmetric metal–organic framework-based mixed matrix membrane for reversible self-assembling 3d architecture. ACS Appl. Polym. Mater. **5**(9), 7090–7097 (2023). 10.1021/acsapm.3c01132

[26] F. Zhu, S. Feng, Z. Wang, Z. Zuo, S. Zhu et al., Co-ion specific effect aided phase separation in polyelectrolyte hydrogels toward extreme strengthening and toughening. Macromolecules **56**(15), 5881–5890 (2023). 10.1021/acs.macromol.2c02583

[27] Q. Zhao, J. Liu, Z. Wu, X. Xu, H. Ma et al., Robust PEDOT:PSS-based hydrogel for highly efficient interfacial solar water purification. Chem. Eng. J. **442**, 136284 (2022). 10.1016/j.cej.2022.136284

[28] X. Cui, Z. Liu, Z. Yi, B. Zhang, X. Gao et al., Reprogrammable soft actuators based on a photochromic organic–inorganic hybrid membrane with modulatable NIR photothermal conversion. J. Colloid Interface Sci. **692**, 137460 (2025). 10.1016/j.jcis.2025.137460 40188794 10.1016/j.jcis.2025.137460

[29] Y. Xue, X. Chen, F. Wang, J. Lin, J. Liu, Mechanically-compliant bioelectronic interfaces through fatigue-resistant conducting polymer hydrogel coating. Adv. Mater. **35**(40), e2304095 (2023). 10.1002/adma.202304095 37381603 10.1002/adma.202304095

[30] P. Zhang, I.M. Lei, G. Chen, J. Lin, X. Chen et al., Integrated 3d printing of flexible electroluminescent devices and soft robots. Nat. Commun. **13**(1), 4775 (2022). 10.1038/s41467-022-32126-1 35999212 10.1038/s41467-022-32126-1 PMC9399151

[31] H. Kim, S.-k Ahn, D.M. Mackie, J. Kwon, S.H. Kim et al., Shape morphing smart 3d actuator materials for micro soft robot. Mater. Today **41**, 243–269 (2020). 10.1016/j.mattod.2020.06.005

[32] X. Wei, Z. Wu, H. Gao, S. Cao, X. Meng et al., Mechano-gated iontronic piezomemristor for temporal-tactile neuromorphic plasticity. Nat. Commun. **16**(1), 1060 (2025). 10.1038/s41467-025-56393-w 39865134 10.1038/s41467-025-56393-w PMC11770186

[33] Z. Zhao, Z. Cao, Z. Wu, W. Du, X. Meng et al., Bicontinuous vitrimer heterogels with wide-span switchable stiffness-gated iontronic coordination. Sci. Adv. (2024). 10.1126/sciadv.adl2737 10.1126/sciadv.adl2737 PMC10923496 38457508

[34] S.-i Takata, K. Suzuki, T. Norisuye, M. Shibayama, Dependence of shrinking kinetics of poly(N-isopropylacrylamide) gels on preparation temperature. Polymer **43**(10), 3101–3107 (2002). 10.1016/S0032-3861(02)00089-7

[35] Y. Hirokawa, H. Jinnai, Y. Nishikawa, T. Okamoto, T. Hashimoto, Direct observation of internal structures in poly(N-isopropylacrylamide) chemical gels. Macromolecules **32**(21), 7093–7099 (1999). 10.1021/ma990437v

[36] X. Cui, Z. Liu, B. Zhang, X. Tang, F. Fan et al., Sponge-like, semi-interpenetrating self-sensory hydrogel for smart photothermal-responsive soft actuator with biomimetic self-diagnostic intelligence. Chem. Eng. J. **467**, 143515 (2023). 10.1016/j.cej.2023.143515

