# Supplementary material for: High-Strength, Self-Sensing Multiphase Hydrogels for Load-Bearing Actuation and Logical Human–Machine Interaction
Source: Nanomicro Lett. 2026 Jul 23;18:450. doi: 10.1007/s40820-026-02280-y (PMC13391480; doi:10.1007/s40820-026-02280-y)
Supplement: Supplementary file 6 — (DOCX 28142 KB) [file 40820_2026_2280_MOESM6_ESM.docx]

Supporting Information for

**High-Strength, Self-Sensing Multiphase Hydrogels for Load-Bearing Actuation and Logical Human–Machine Interaction**

Zhilin Zhang^1^, Jiayi Gu^1^, Lina Wang^2^, Xingchen Cui^1^, Xu Zhai^4^, Yue Xu^1^, He Liu^2^, Deliang Li^2^, Bingle Li^2^, Ye Tian^2,^ *, Baoyang Lu^3,^ *, Yu Fu^1, 4,^ * and Tieqiang Wang^1,^ *

^1^ Department of Chemistry, College of Sciences, Northeastern University, Shenyang 110819, P. R. China

^2^ College of Medicine and Biological Information Engineering, Northeastern University, Shenyang 110169, P. R. China

^3^ Jiangxi Province Key Laboratory of Flexible Electronics, Jiangxi Science and Technology Normal University, Jiangxi Science & Technology Normal University, Nanchang, 330013, P. R. China

^4^ School of Chemical and Environmental Engineering, Sichuan University of Science and Engineering, Zigong 643000, P. R. China

*Corresponding authors. E-mail: [wangtieqiang@mail.neu.edu.cn](mailto:wangtieqiang@mail.neu.edu.cn) (Tieqiang Wang); [tianye@bmie.neu.edu.cn](mailto:tianye@bmie.neu.edu.cn) (Ye Tian); [luby@jxstnu.edu.cn](mailto:luby@jxstnu.edu.cn) (Baoyang Lu); [fuyu@mail.neu.edu.cn](mailto:fuyu@mail.neu.edu.cn) (Yu Fu)

**Supplementary Figures and Tables**

**
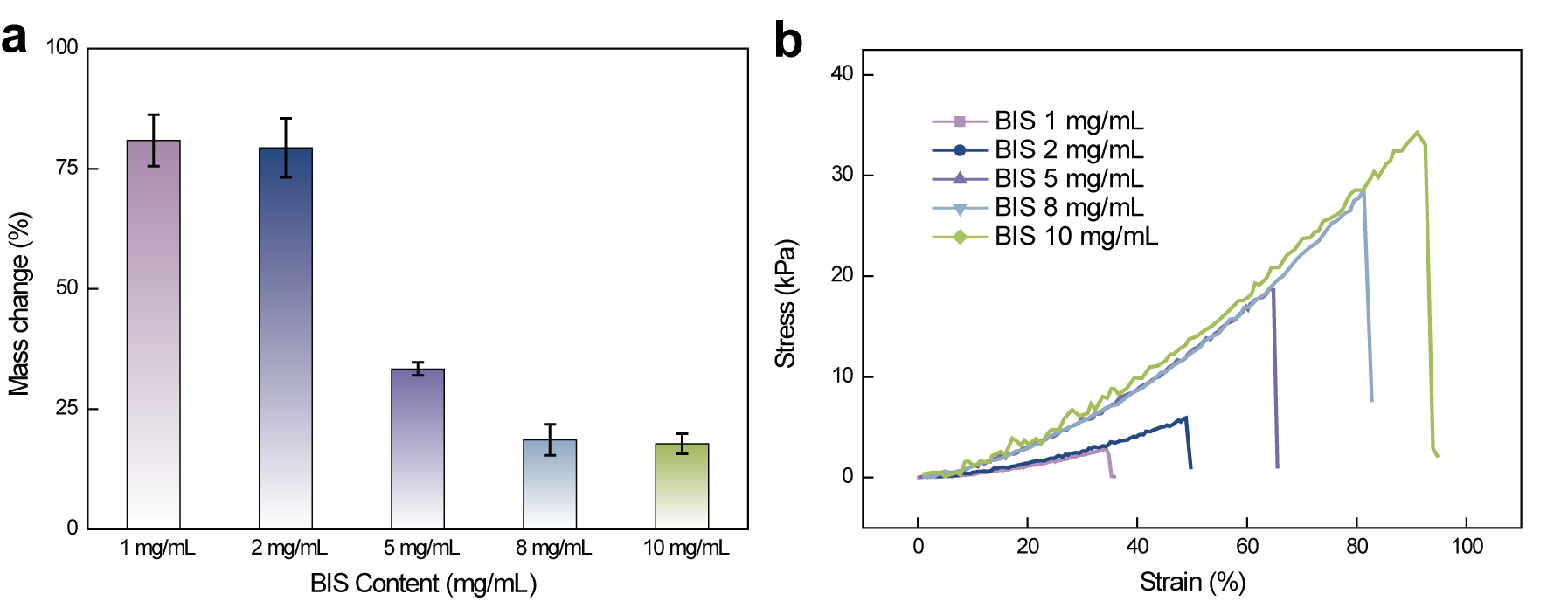
**

**Fig. S1** Properties of pure PNIPAM hydrogels with different crosslinker contents. **a** The ratio of mass of the hydrogel to that of the initial hydrogel when the water is dehydrated to equilibrium at 50 ℃. **b** Stress-strain curves. Therefore, without specific notes, PPS hydrogels with 2 mg/mL BIS content are employed in light of its optimal deswelling and swelling kinetics and mechanical performances for the following characterizations and applications.

**
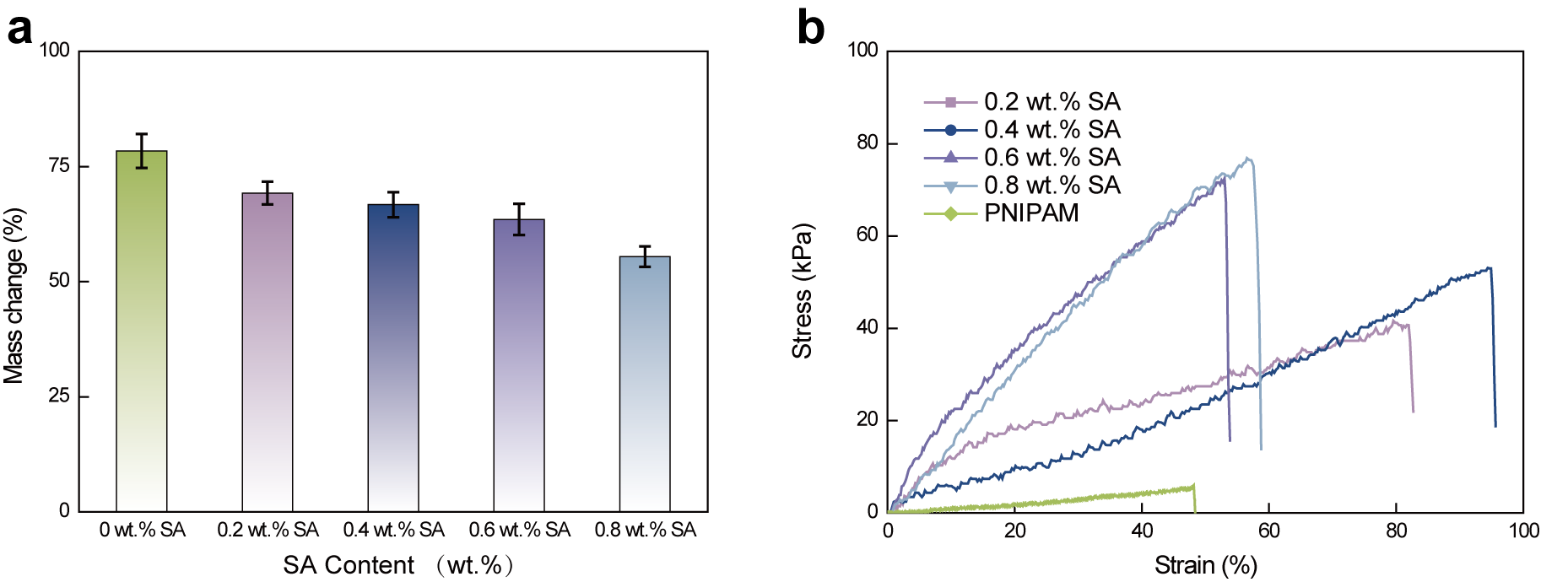
**

**Fig. S2** The performance was optimized by immersion in sodium alginate solutions of different concentrations and cross-linking in calcium chloride for 60 s (*i.e.*, 0, 0.2, 0.4, 0.6 and 0.8 *wt.*%). **a** The ratio of mass of the hydrogel to that of the initial hydrogel when the water is dehydrated to equilibrium at 50 ℃. **b** Stress-strain curves. Therefore, without specific notes, PPS hydrogels with 0.6 *wt.*% content are employed in light of its optimal deswelling and swelling kinetics and mechanical performances for the following characterizations and applications.

**
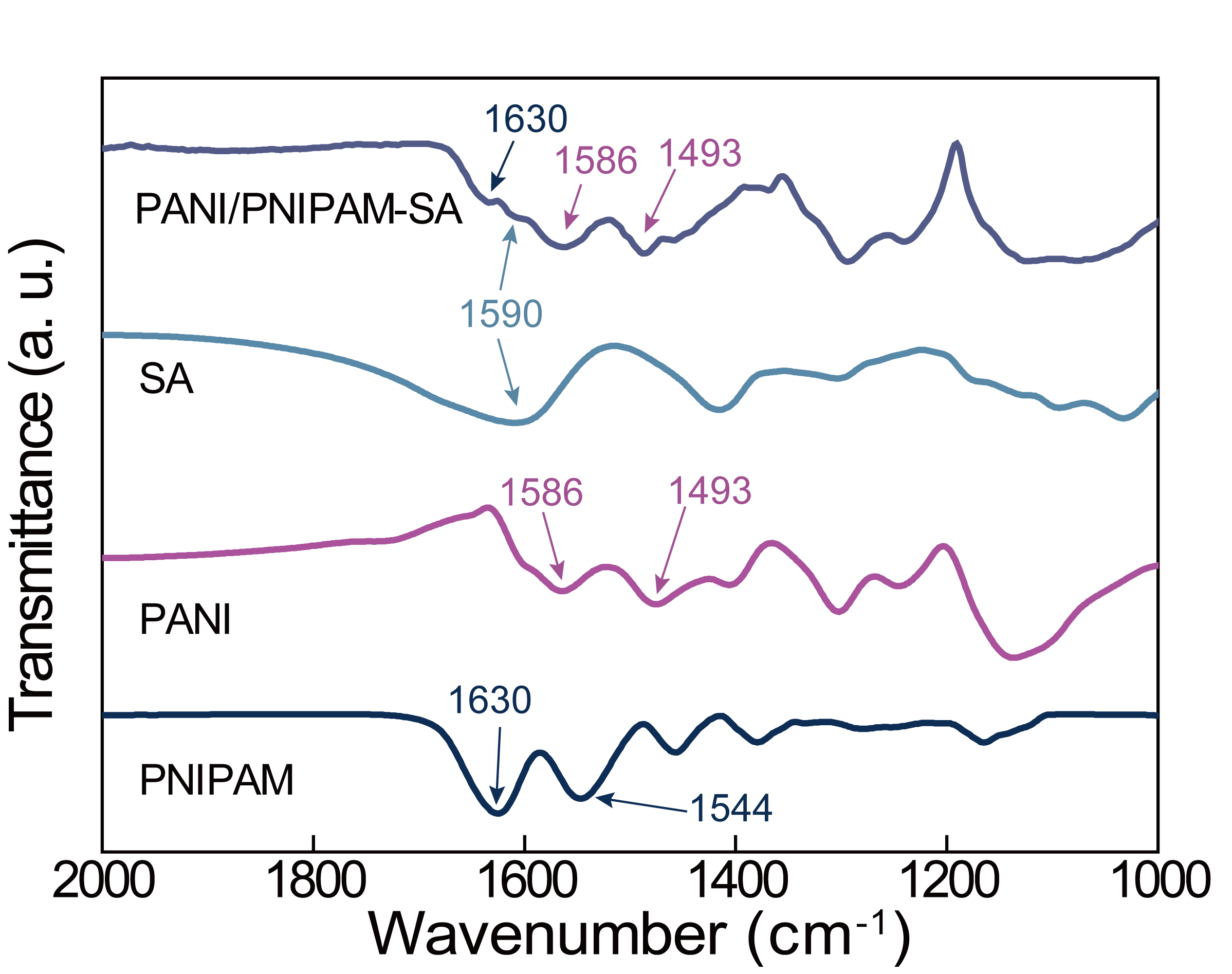
**

**Fig. S3** FT-IR spectra of PNIPAM, PANI, SA and PANI/PNIPAM-SA samples. PANI/PNIPAM-SA combines the characteristic peaks of PANI, PNIPAM and SA.


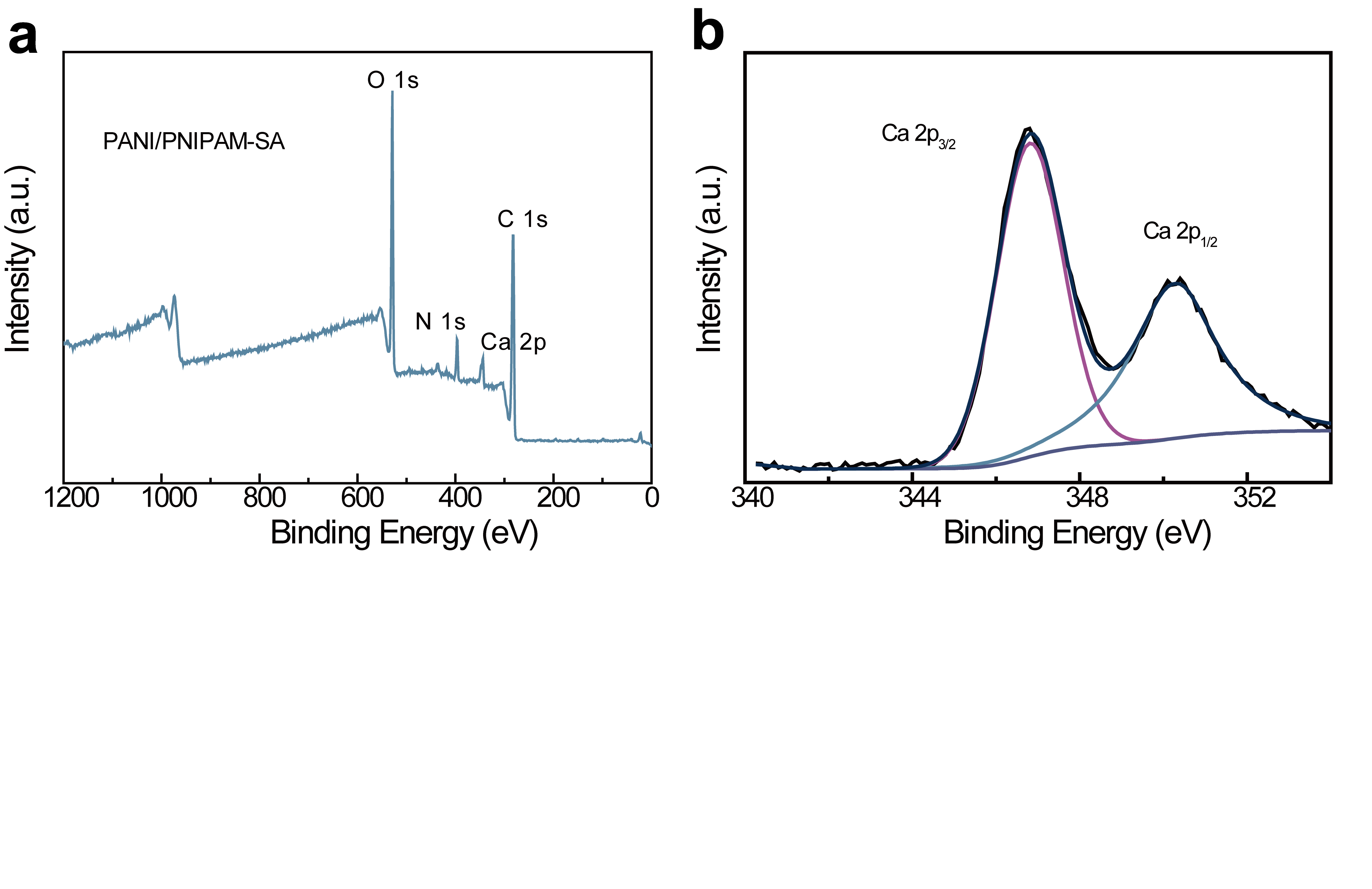


**Fig. S4 a** XPS Spectra of PPS Hydrogel. **b** High resolution XPS spectra of PPS hydrogel Ca^2+^.


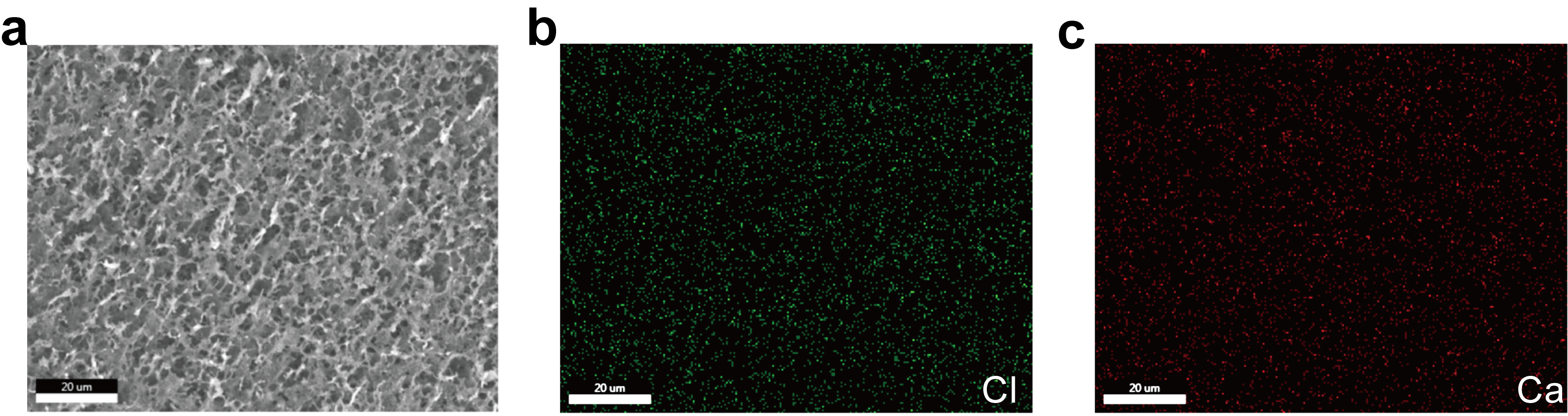


**Fig. S5** SEM image and EDS mapping image of PPS hydrogel. **a** SEM image of PPS hydrogel. Distribution map of **b** Cl and **c** Ca elements of PPS hydrogel.

**
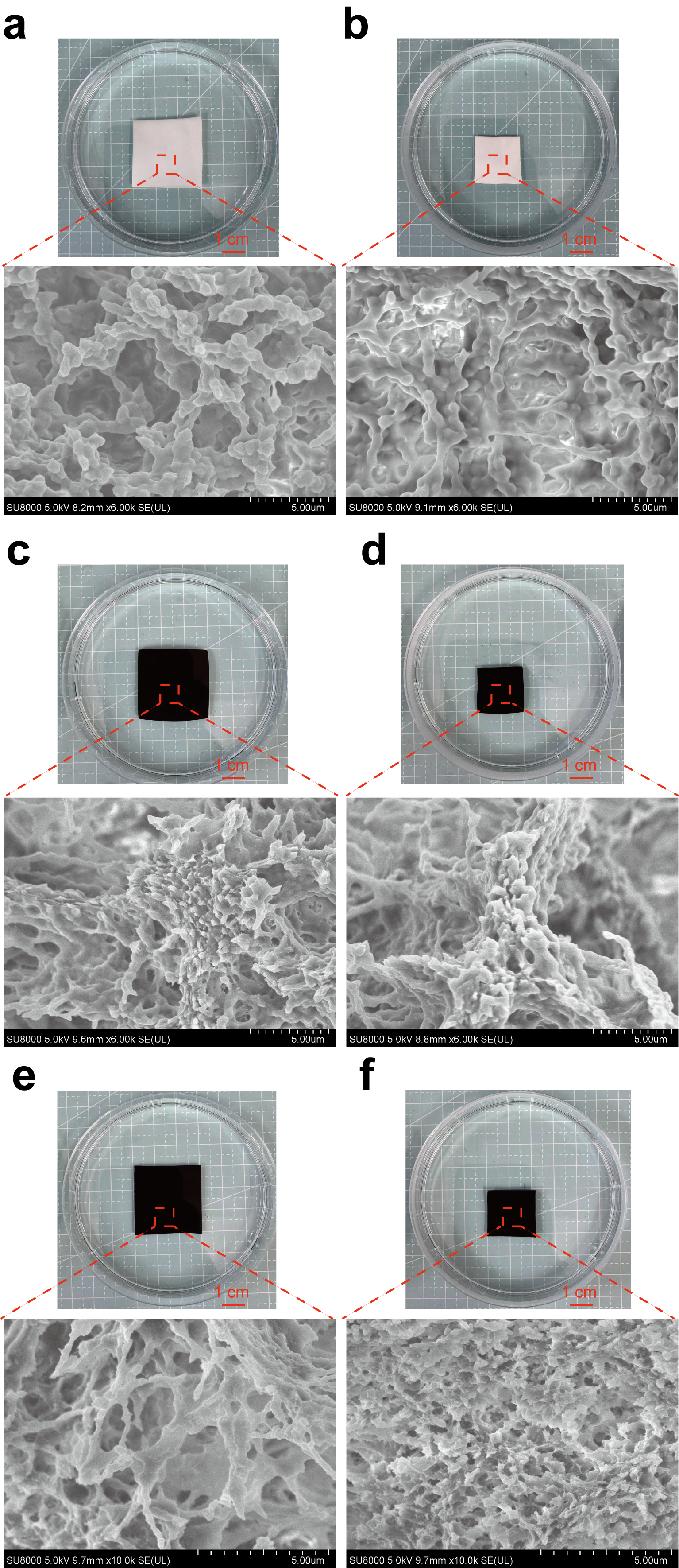
**

**Fig. S6** Photographs and cross-sectional SEM images of hydrogels at different temperatures. **a** PNIPAM swelling at 20 °C. **b** PNIPAM deswelling at 50 °C. **c** PANI/PNIPAM swelling at 20 °C. **d** PANI/PNIPAM deswelling at 50 °C. **e** PANI/PNIPAM-SA swelling at 20 °C. **f** PANI/PNIPAM-SA deswelling at 50 °C.

**
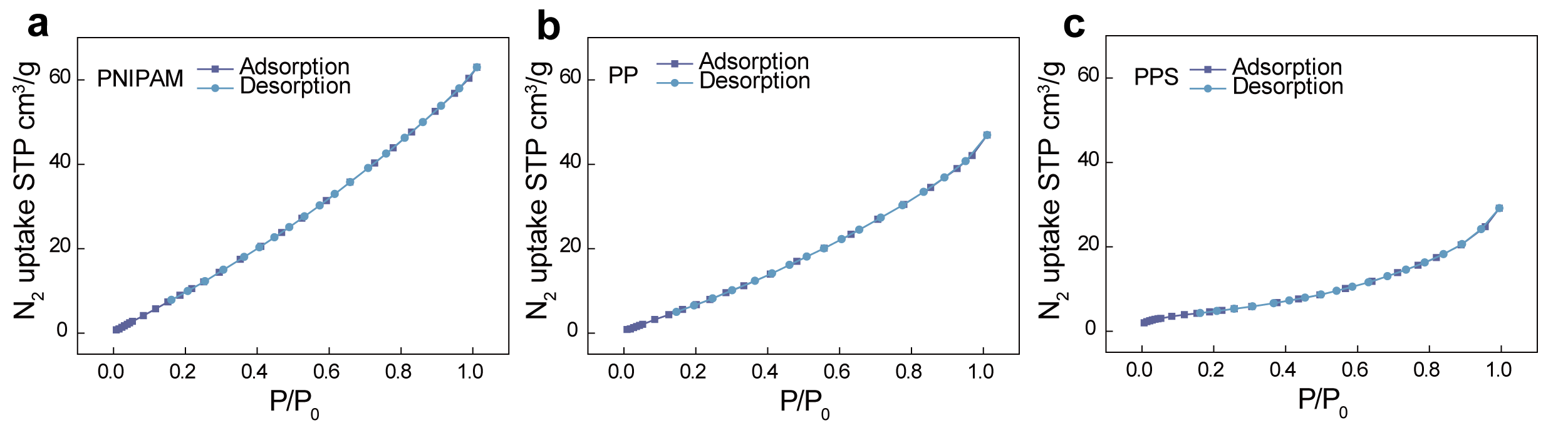
**

**Fig. S7** N_2_ adsorption-desorption isotherms of **a** PNIPAM hydrogel, **b** PP and **c** PPS hydrogel.


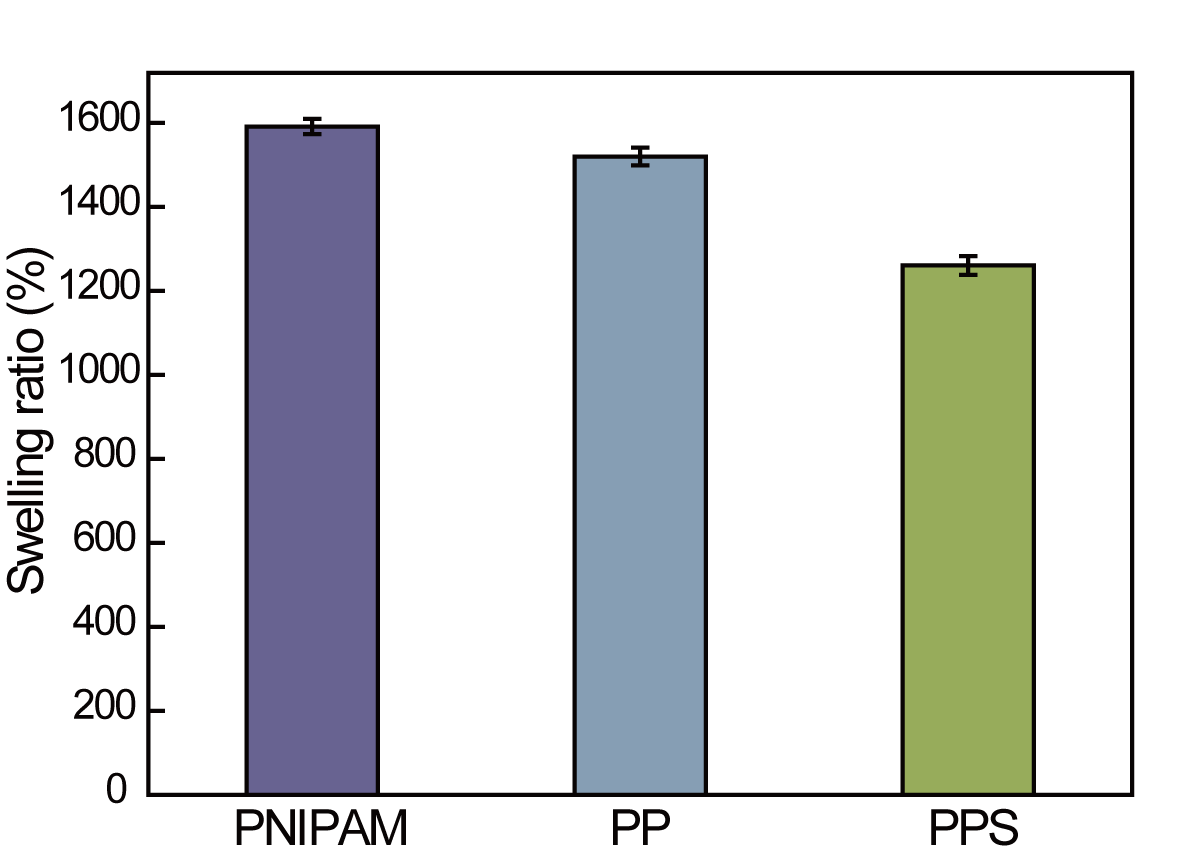


**Fig. S8** Equilibrium swelling rate of PNIPAM, PP, PPS hydrogels.


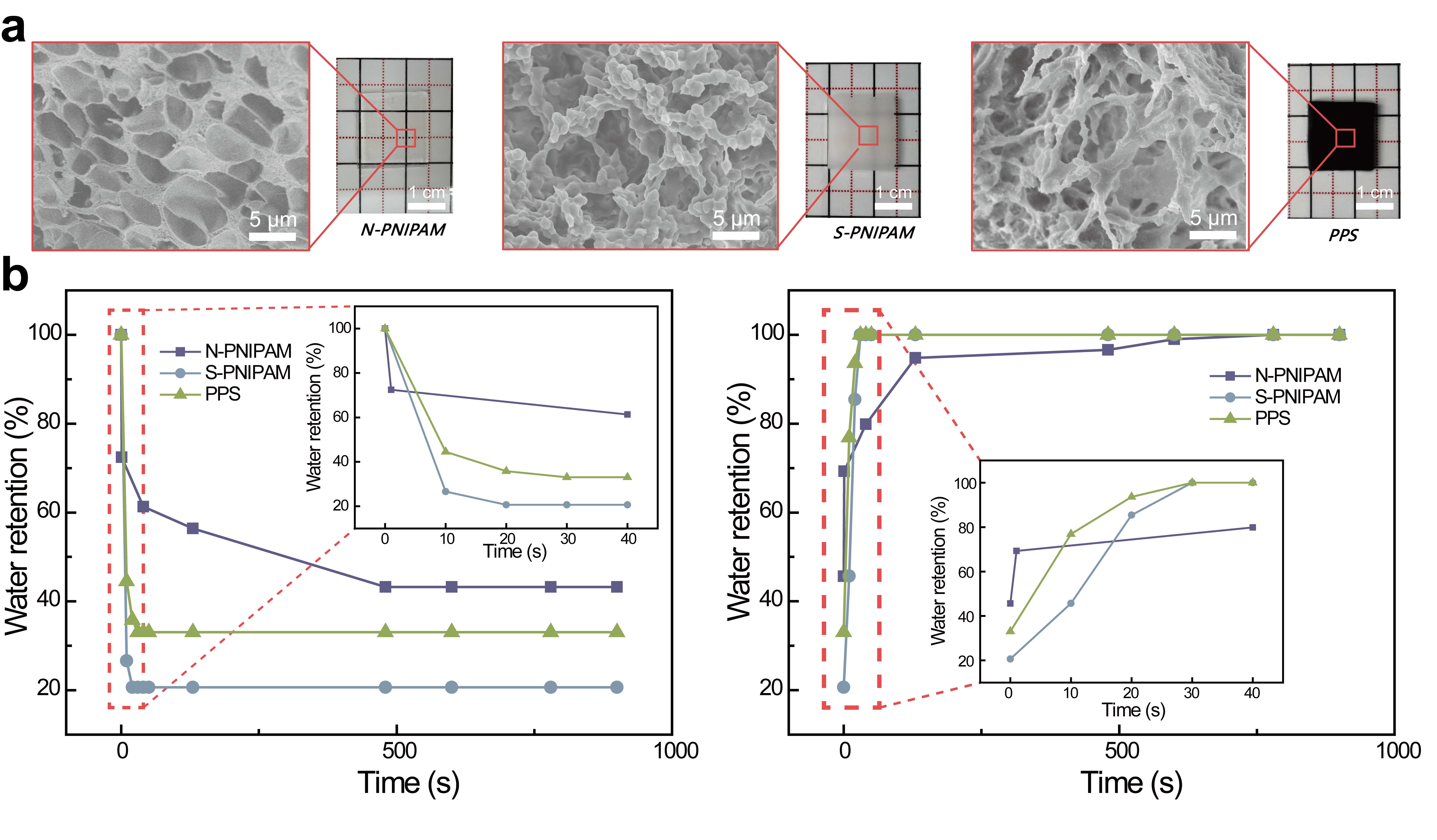


**Fig. S9** Rapid photothermal response mechanism. **a** SEM images (left) and optical images (right) of normal PNIPAM (N-PNIPAM) hydrogels, sponge-like PNIPAM (S-PNIPAM) hydrogels and PPS hydrogel. **b** Deswelling kinetics of N-PNIPAM, S-PNIPAM and PPS hydrogel. F Swelling kinetics of N-PNIPAM, S-PNIPAM and PPS hydrogel.


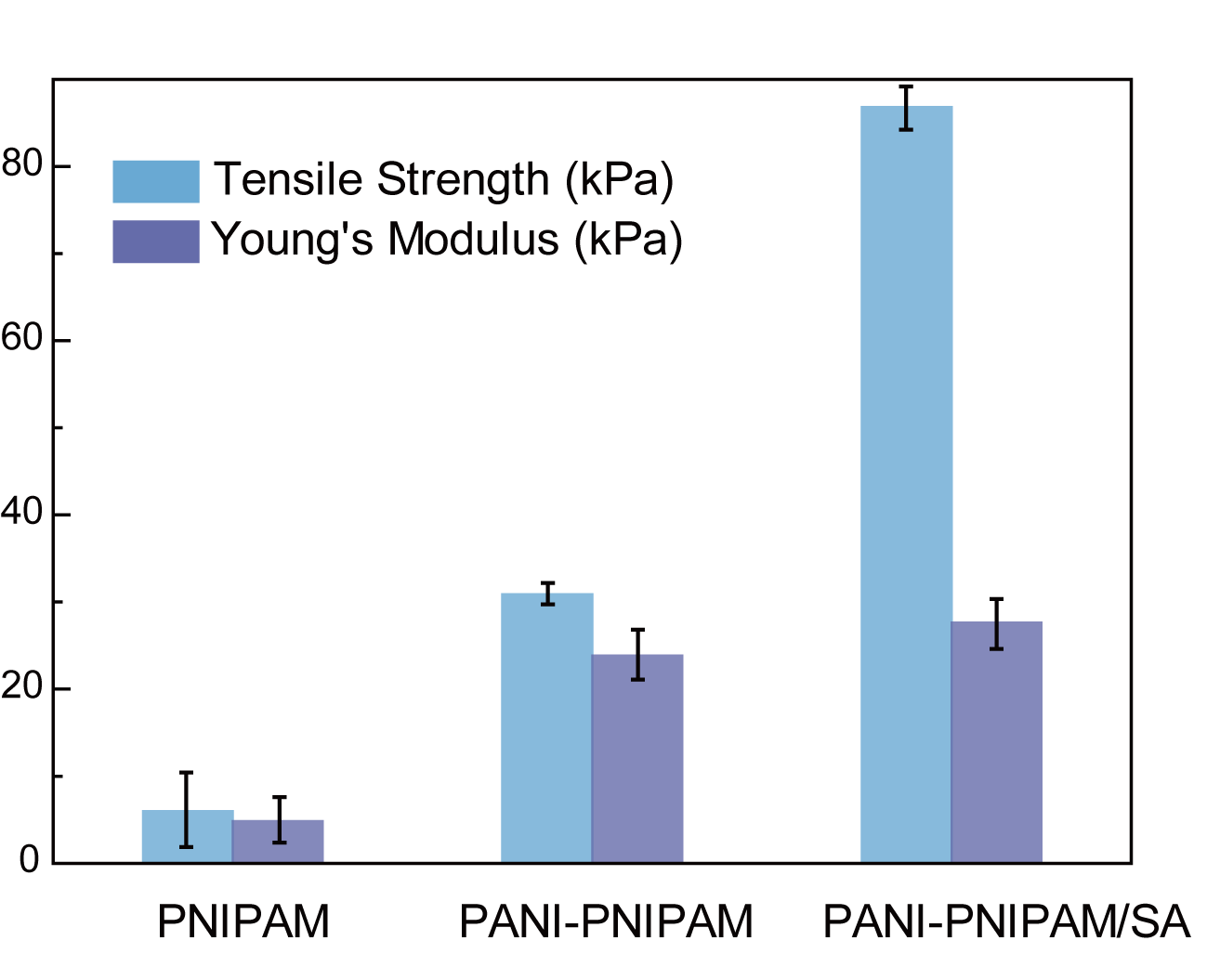


**Fig. S10** Mechanical properties of hydrogels at different stages of material preparation. *i.e.*, PNIPAM, PANI-PNIPAM (PP) and PANI-PNIPAM/SA (PPS). Tensile strength and Young’s modulus.


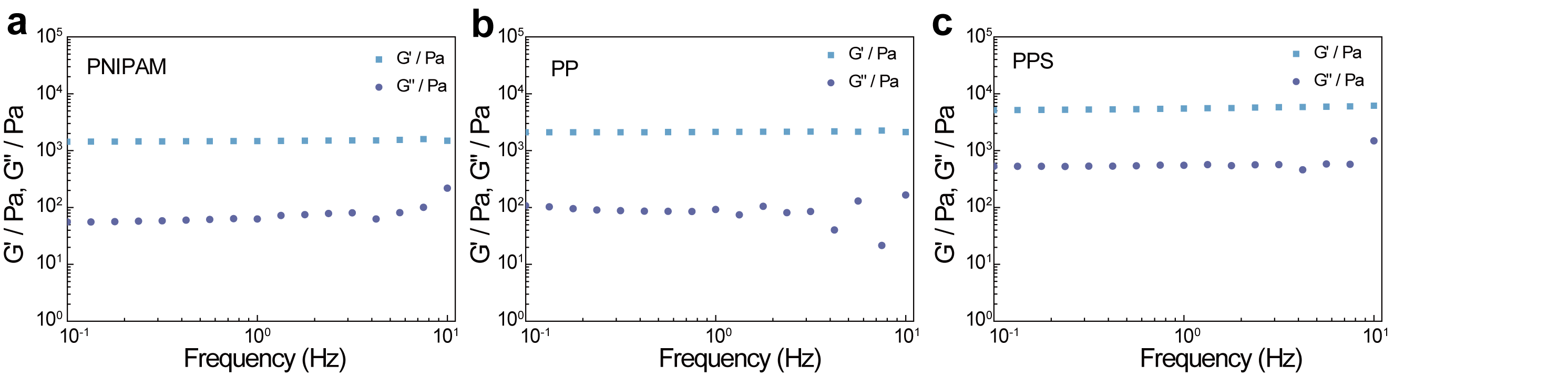


**Fig. S11** Rheological test of **a** PNIPAM, **b** PP, **c** PPS hydrogels.


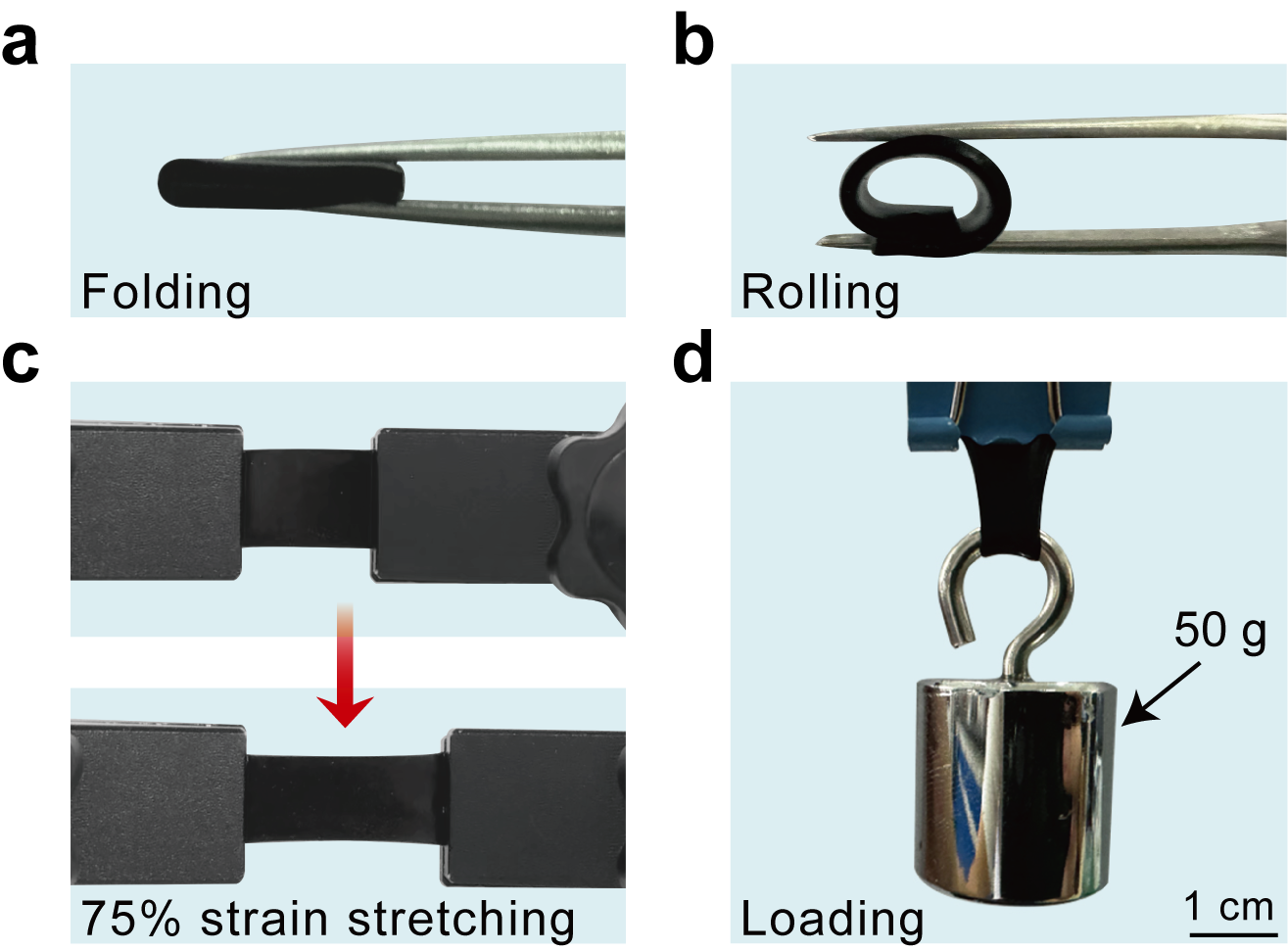


**Fig. S12** Flexible and high-strength optical images of PPS hydrogel. Image of **a** folding, **b** rolling, **c** stretching the PPS hydrogels, displaying its soft and flexible essence. Such PPS hydrogel also exhibits remarkably **d** high strength, by sustaining a weight (50 g) with over 2500 of its own weight.


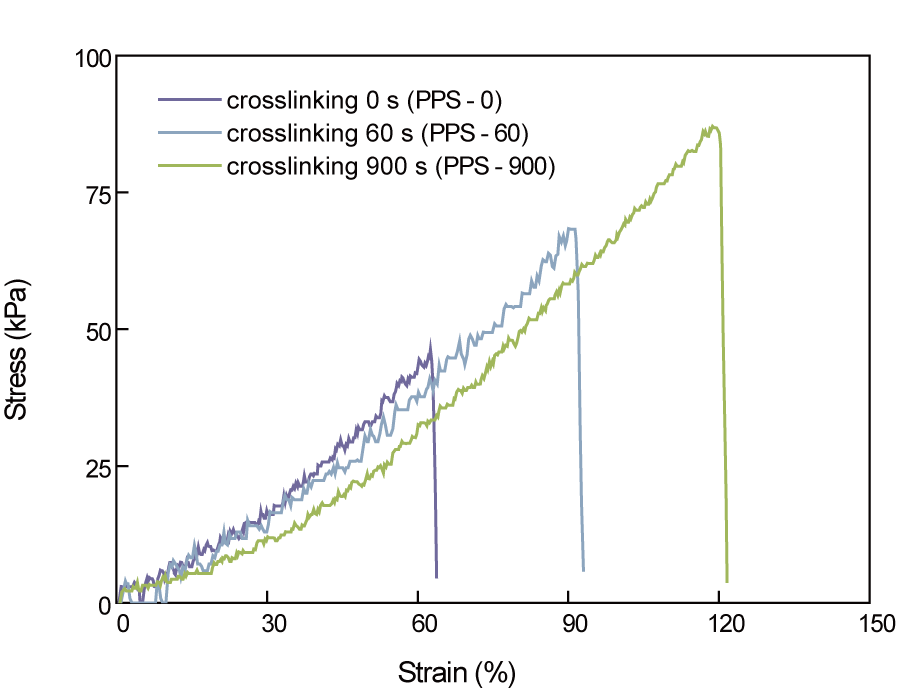


**Fig. S13** Mechanical properties of PPS hydrogels cross-linked for different times in calcium chloride solution.

**
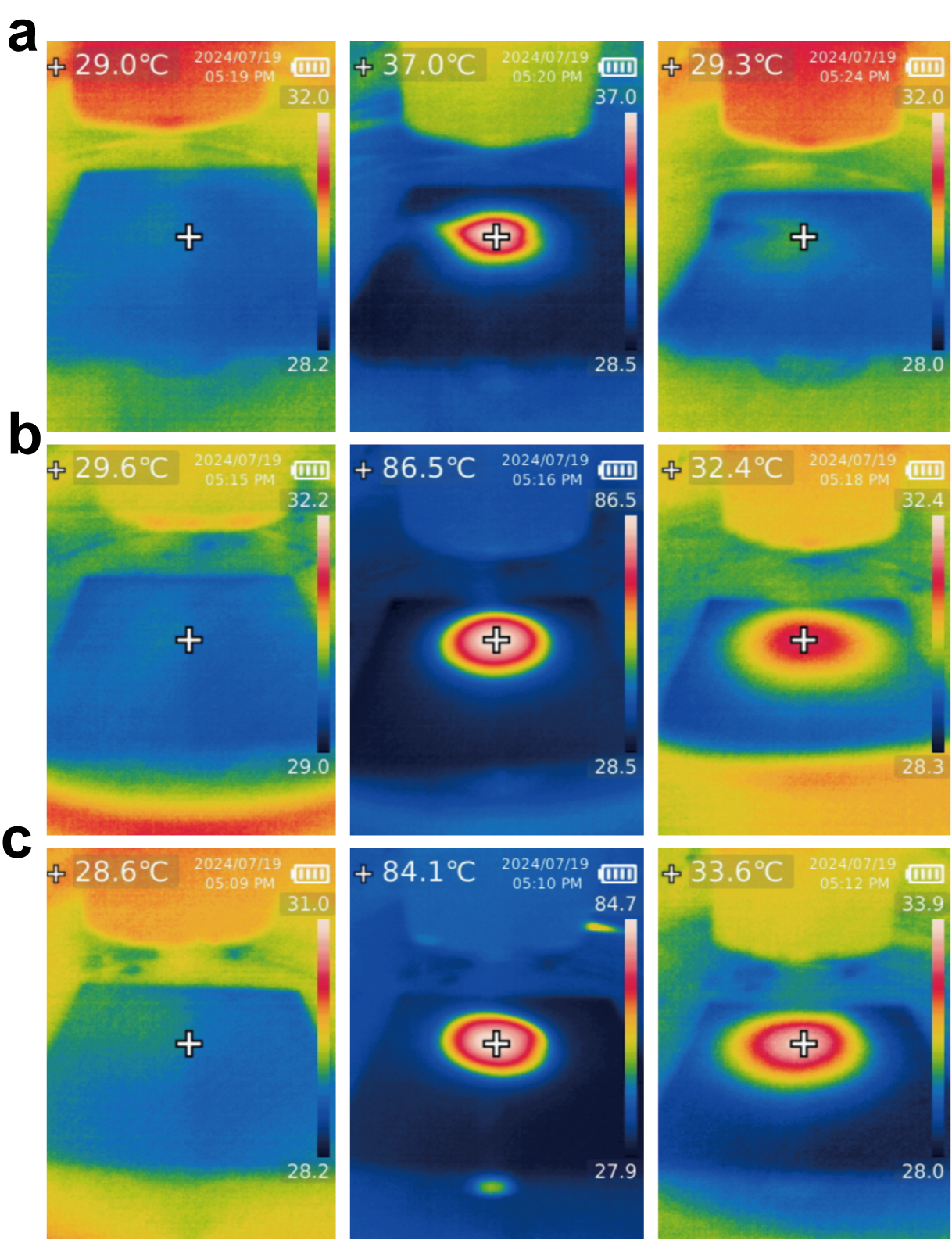
**

**Fig. S14** Temperature changes of hydrogel under near-infrared light under infrared thermal imaging camera. **a** PNIPAM, **b**) PP, **c** PPS. During the on and off process of NIR light, the intensity of NIR light is 1.6 w cm^-2^.


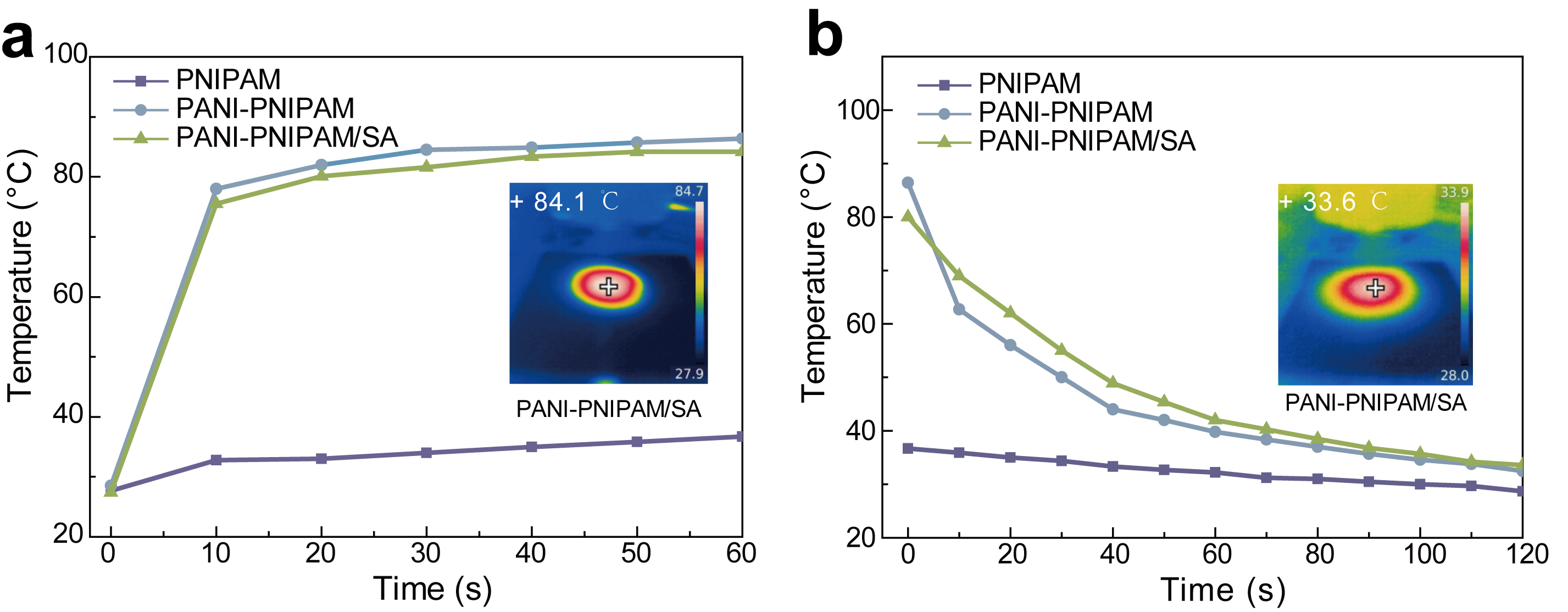


**Fig. S15** Photothermal performance of PNIPAM, PANI-PNIPAM and PANI-PNIPAM/SA. **a** Temperature change of hydrogel through the on process of NIR light with 1.6 w cm^-2^. **b** Dynamic surface temperature of hydrogel cooling in air.


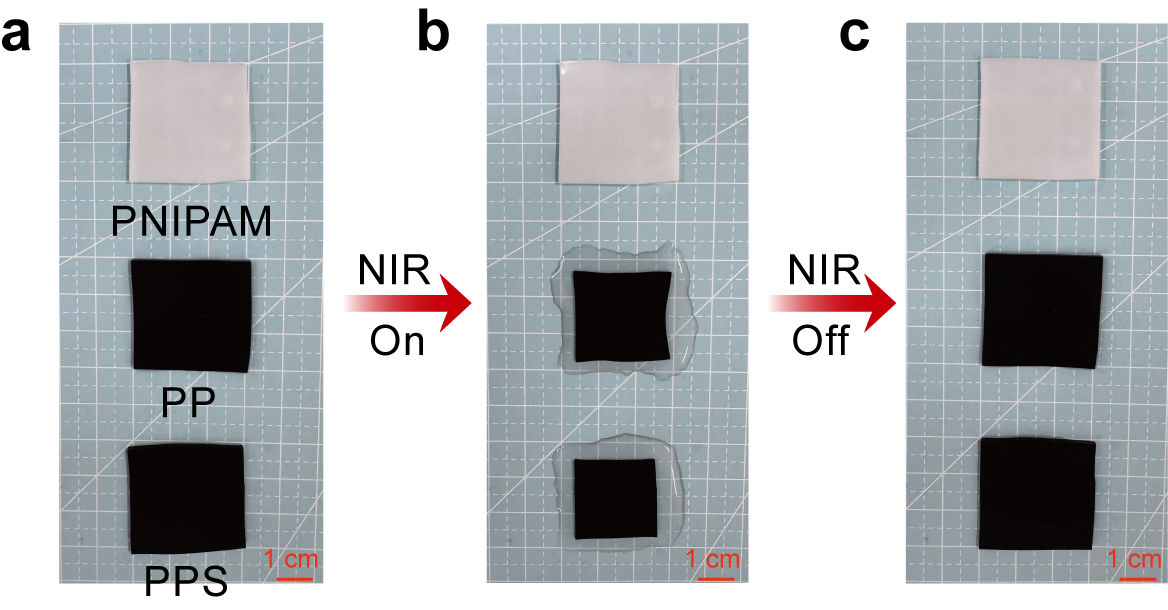


**Fig. S16** The volume change of water gel under near infrared light. **a** The original state of the hydrogel. **b** NIR on. **c** NIR off.


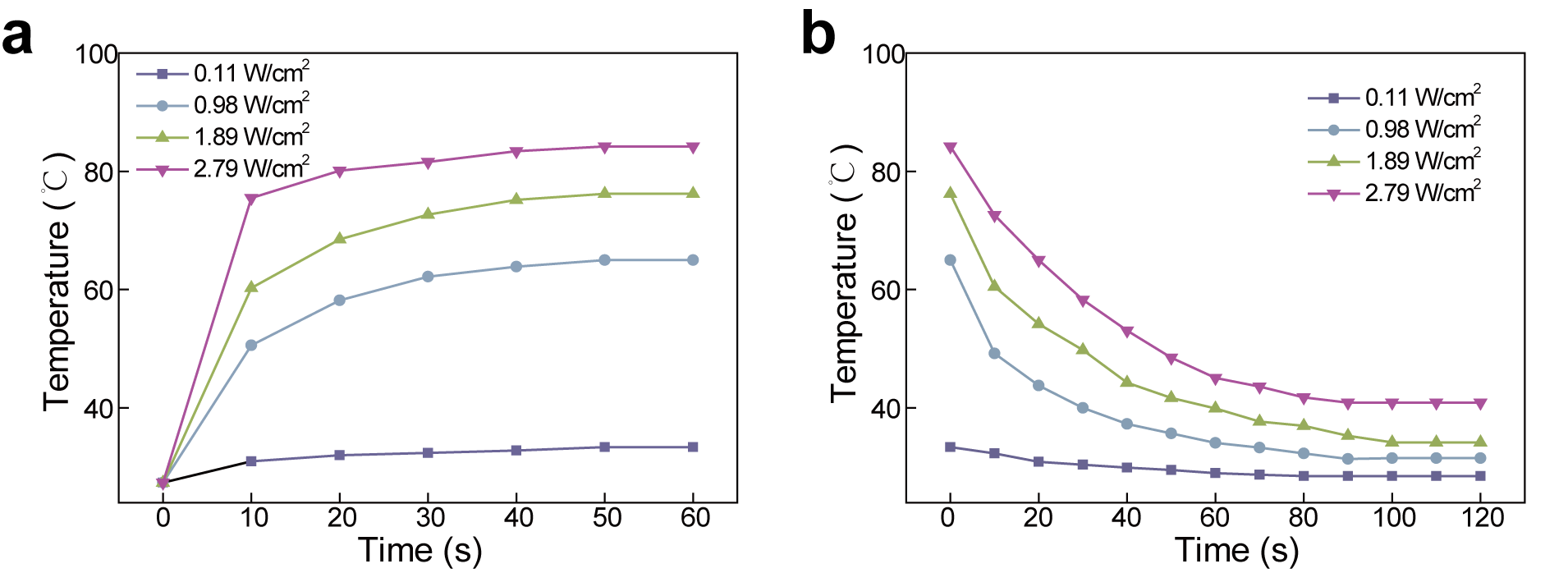


**Fig. S17** Photothermal performance of PPS hydrogel. **a** Dynamic surface temperature of PPS exposed to NIR light with different intensity; **b** Dynamic surface temperature of PPS cooling in air.


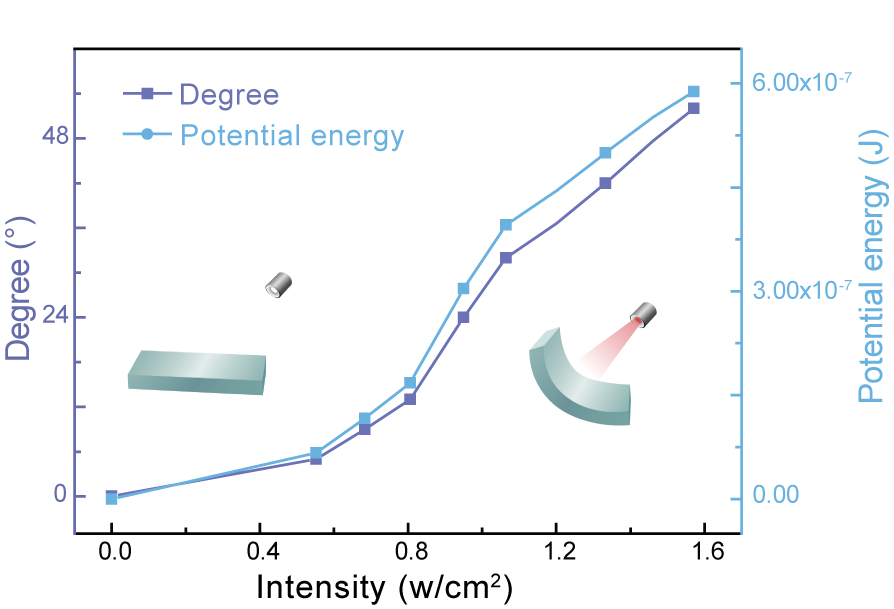


**Fig. S18** Irradiation intensity dependent folding angle (left Y-axis) and gravitational potential energy increment (right Y-axis) of PPS hydrogel soft actuator.

**
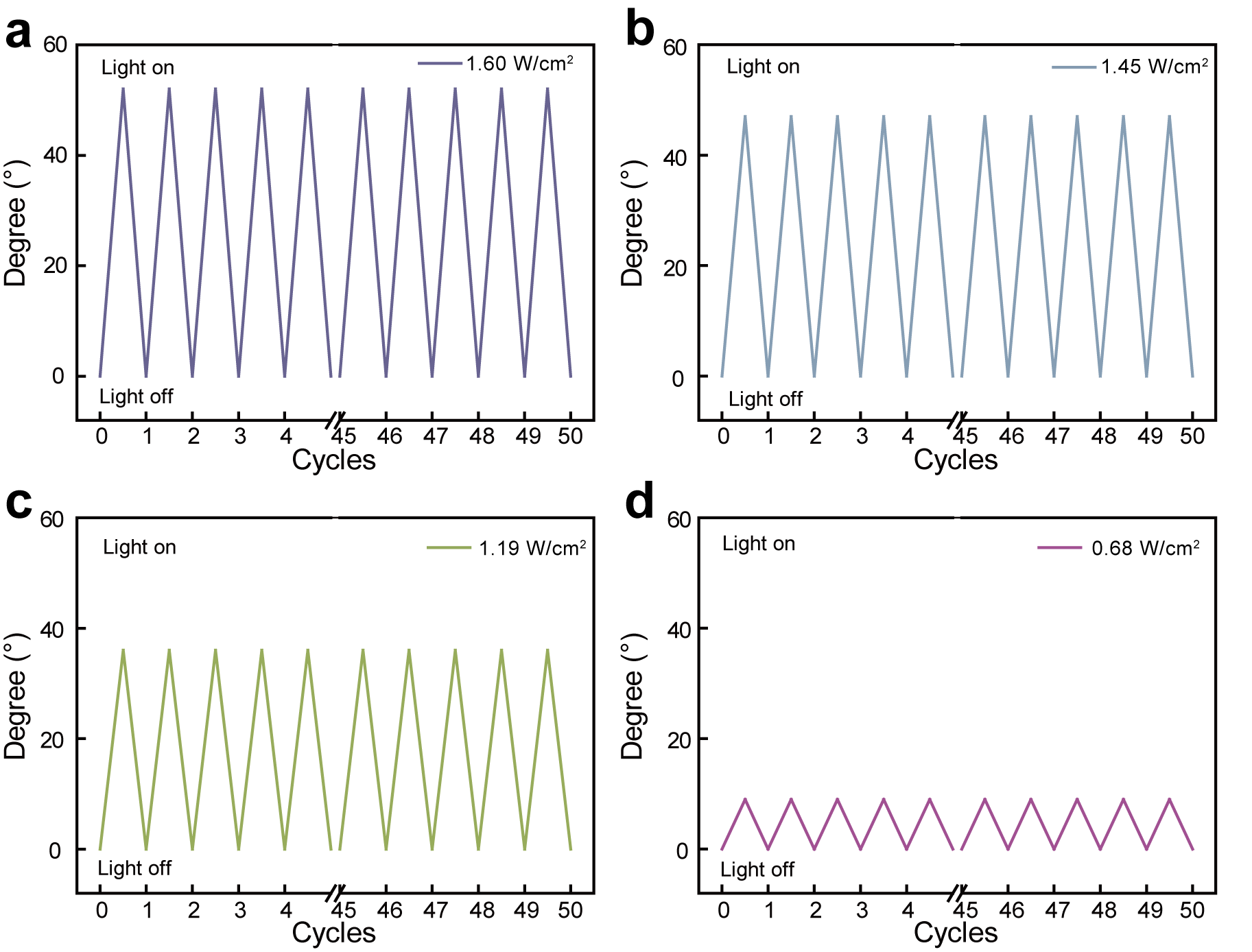
**

**Fig. S19** Bending angle and stability of PPS hydrogel after 50 switching cycles under near-infrared light of different powers. **a** 1.6 W cm^-2^, **b** 1.45 W cm^-2^, **c** 1.19 W cm^-2^, **d** 0.68 W cm^-2^.


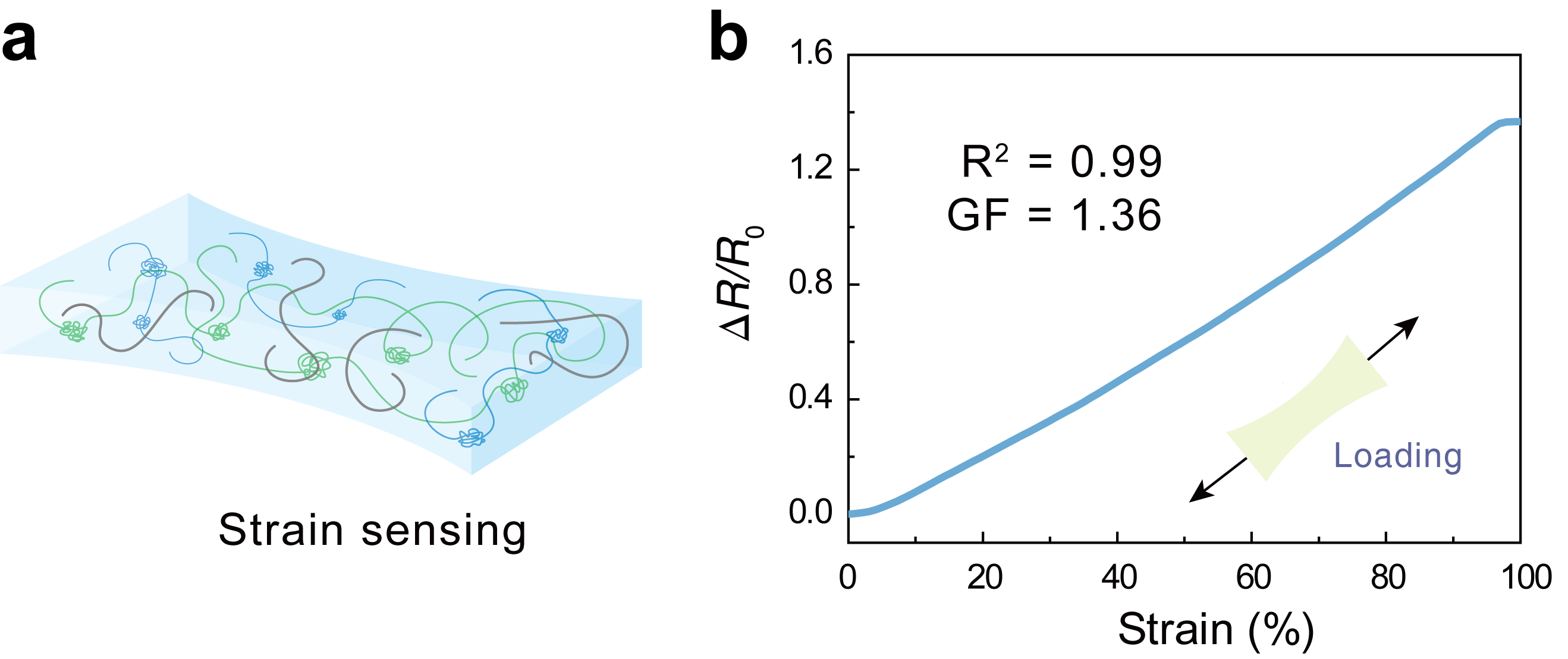


**Fig. S20** Sensing performance of PPS hydrogel. **a** Schematic illustration of hydrogel stretching. **b** The relative resistance change of PPS hydrogel versus strain.


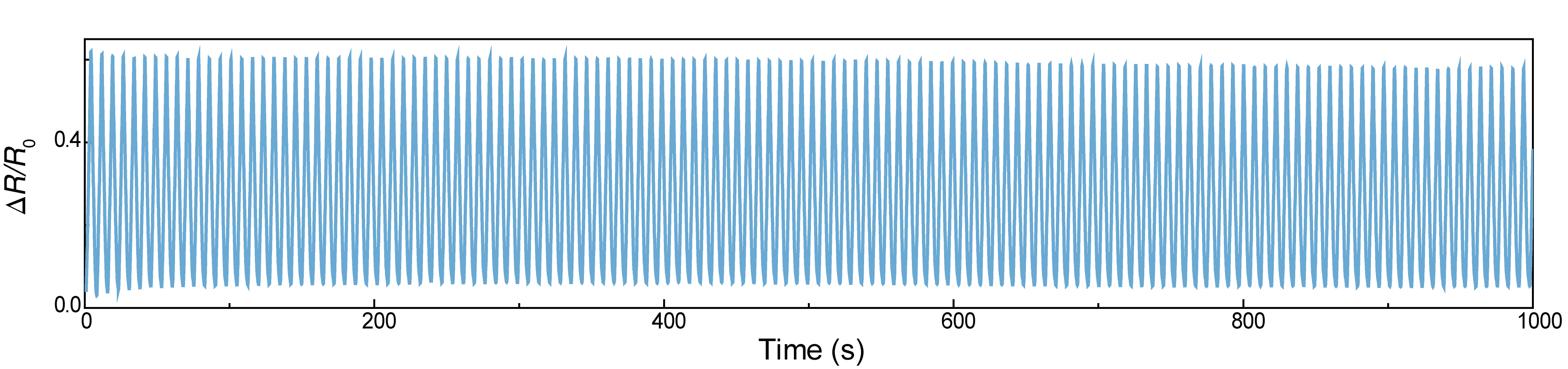


**Fig. S21** The relative resistance change of PPS hydrogels under 100% strain for 200 cycles.

**
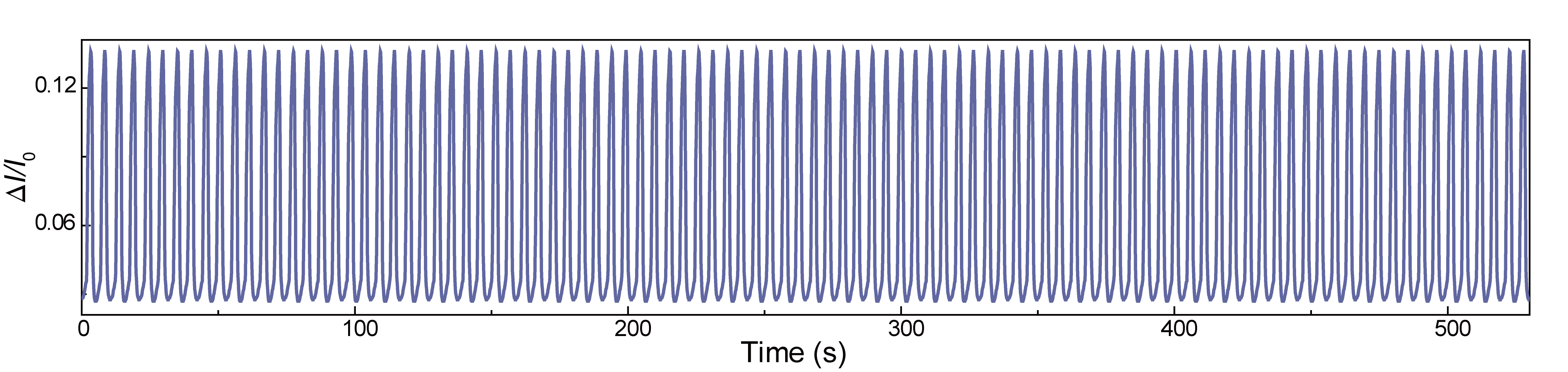
**

**Fig. S22** Electrical monitoring signal by PPS hydrogel actuator under the NIR light with 1.60 w cm^-2^ for 200 cycles.

**
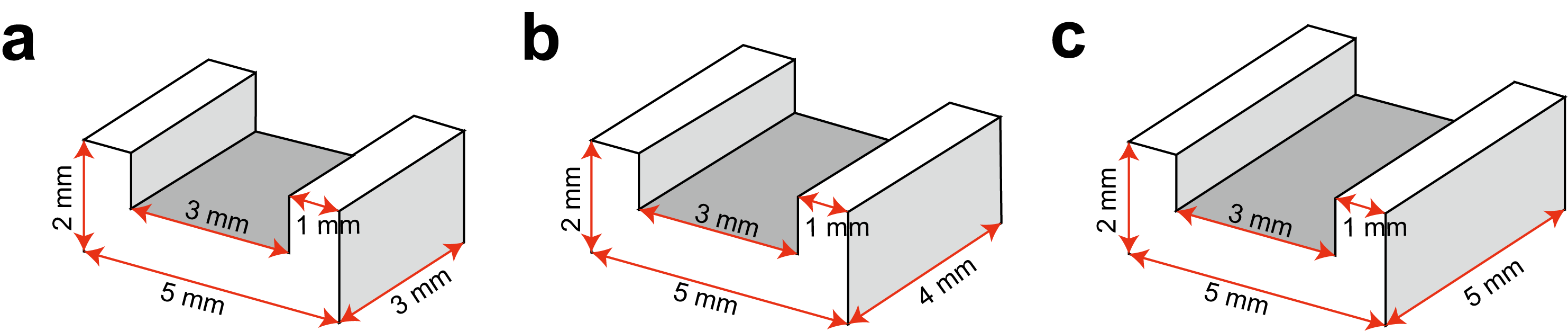
**

**Fig. S23** 3D printing load schematic diagram. **a** m_b_, **b** m_c_, **c** m_d_.


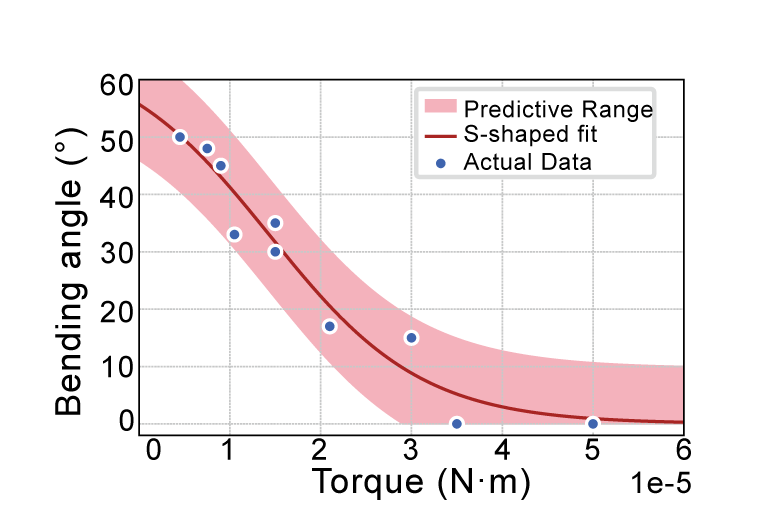


**Fig. S24** The bending angle of PPS-900 hydrogel under different loads and different torques.

**
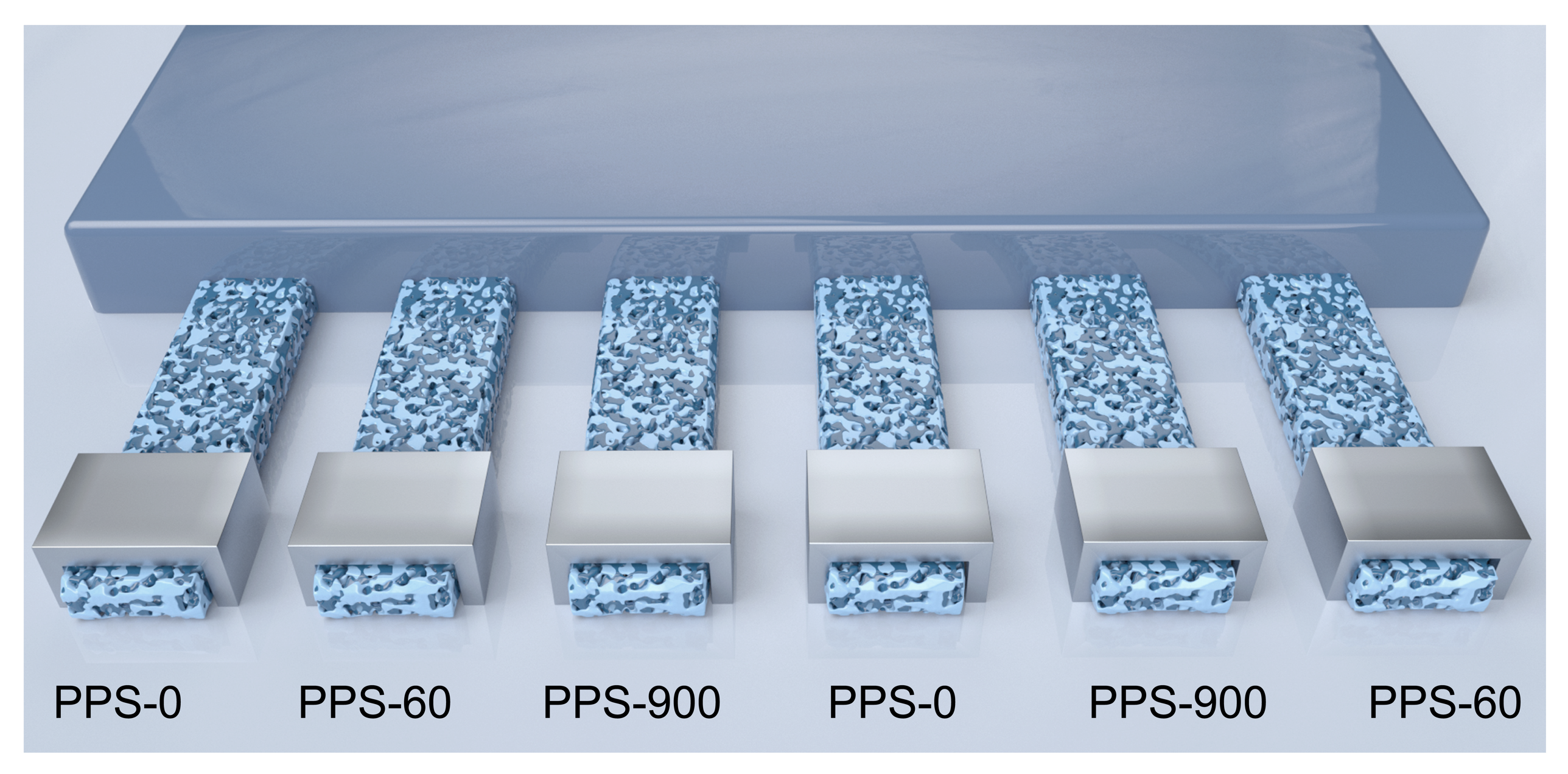
**

**Fig. S25** Hydrogel array for manufacturing logic gate information encryption system. The hydrogel is PPS-0, PPS-60, PPS-900, PPS-0, PPS-900, PPS-60 from left to right. The diversity of passwords can be reflected by changing the arrangement order of materials.


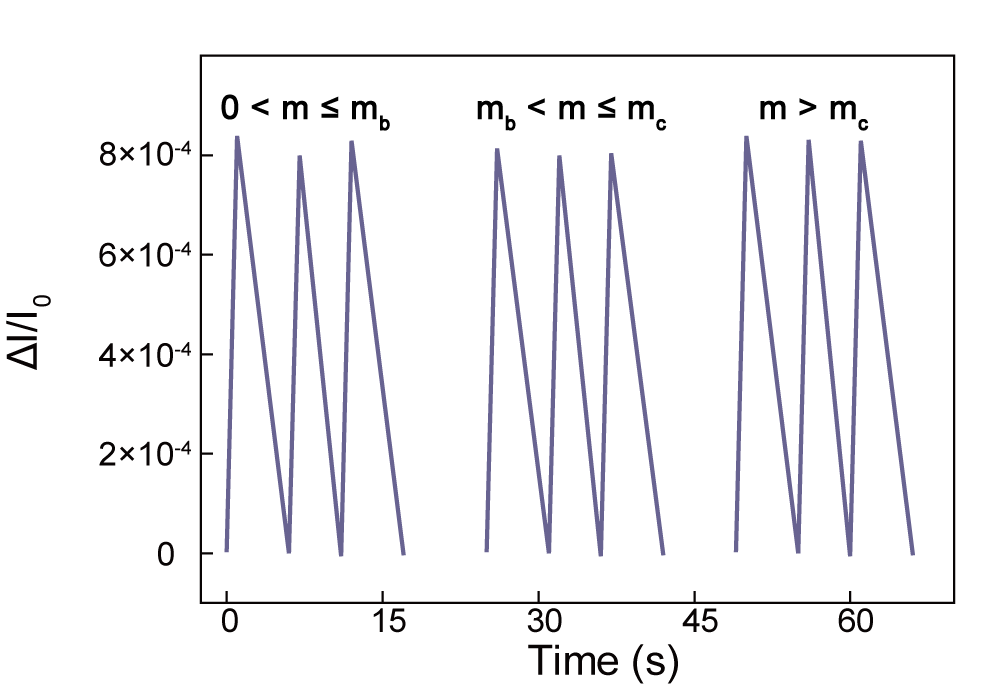


**Fig. S26** Electrical signals of PPS-0 hydrogel under loading conditions.

**Table S1** Comparison of the PANI/PNIPAM-SA hydrogel actuator in this work with previously reported PNIPAM hydrogels-based actuator

| **Materials** | **Response time (s)** | **Recover time (s)** | **Tensile strength (kPa)** | **Ref.** |
| --- | --- | --- | --- | --- |
| K-MXene/PEDOT:PSS-PNIPAM | 11 | 19 | 12 | *Adv. Funct. Mater.* **2023**, 33, 2214867. |
| CNTs-Ecoflex/PNIPAm | 25 | 105 | 12 | *Chem. Eng. J.* **2021**, 415, 128988. |
| MoO_2_/ PNIPAM | 30 | 70 | 25 | *ACS Appl. Mater. Interfaces* **2021**, 13, 28, 33404–33416. |
| PDA@MXene/PNIPAM | 15 | - | 27 | *Sensor Actuat B-chem.* **2023**, 390,133899. |
| PNIPAM/TOCN/PAM | 60 | 600 | 24 | *Carbohyd. Polym.* **2024**, 335, 122067. |
| MXene/PNIPAM | 10 | - | 81.88 | *Adv. Funct. Mater.* **2023**, 33, 2301982. |
| PNIPAM-nSiO_2_ | 60 | 120 | 23 | *Macromol. Res.* **2023**, 31, 625–633. |
| PNIPM/PANI | 15 | 600 | 20.7 | *J. Mater. Chem. C,* **2023**, 11, 6741–6749. |
| PNIPM/PANI | 2.5 | 20 | 18 | *Sci. Robot.* **2021**, 6, eabd5483. |
| PANI/PNIPAM -SA | 7.57 | 5.04 | 86.83 | Our work |

**Table S2** Input table showing binary signals denoted by the presence (Yes = 1) or absence (No = 0) of load actuation

| Logic Gate | Input | | | Output |
| --- | --- | --- | --- | --- |
|  | PPS-0 | PPS-60 | PPS-900 |  |
| Yes/No | 1 | 1 | 1 | 0 |
|  | 0 | 1 | 1 | 0 |
|  | 0 | 0 | 1 | 1 |
|  | 0 | 0 | 0 | 0 |
|  | 1 | 1 | 0 | 0 |
|  | 1 | 0 | 0 | 0 |
|  | 0 | 1 | 0 | 0 |
|  | 1 | 0 | 1 | 0 |

// --- 1. Initialization and Parameter Setup ---

DEFINE CONSTANT THRESHOLD = 0.04 // Set the relative current change threshold

// Define sensor interfaces for the three hydrogel channels

DEFINE INTERFACE Sensor_Ch1 // Corresponds to PPS-0

DEFINE INTERFACE Sensor_Ch2 // Corresponds to PPS-90

DEFINE INTERFACE Sensor_Ch3 // Corresponds to PPS-900

// Define the robotic hand controller interface

DEFINE INTERFACE Robotic_Hand

// Initialize the robotic hand to its default straight position

Robotic_Hand.execute_action("Keep_Straight")

// --- 2. Main Control Loop (Continuous Monitoring & Response) ---

START LOOP (Infinite Loop):

// Step A: Signal Acquisition (Read relative current change ΔI/I₀)

VARIABLE current_1 = Sensor_Ch1.read_relative_current()

VARIABLE current_2 = Sensor_Ch2.read_relative_current()

VARIABLE current_3 = Sensor_Ch3.read_relative_current()

// Step B: Comparator Logic (Convert analog signals to binary 0 or 1)

// Default assignment is 0

VARIABLE digital_1 = 0

VARIABLE digital_2 = 0

VARIABLE digital_3 = 0

IF current_1 > THRESHOLD THEN digital_1 = 1

IF current_2 > THRESHOLD THEN digital_2 = 1

IF current_3 > THRESHOLD THEN digital_3 = 1

// Step C: Logic Gate Operation (Corresponds to: Y = NOT A AND NOT B AND C)

// The trigger condition is met ONLY when the binary code is strictly 001

VARIABLE trigger_OK = FALSE

IF (digital_1 == 0) AND (digital_2 == 0) AND (digital_3 == 1) THEN:

trigger_OK = TRUE

END IF

// Step D: Robotic Hand Execution

IF trigger_OK == TRUE THEN:

Robotic_Hand.execute_action("OK_Gesture")

ELSE:

// Covers all other cases like 000, 111, 010, 100, etc.

Robotic_Hand.execute_action("Keep_Straight")

END IF

// Add a short delay to prevent system jitter from high-frequency reading (Optional)

DELAY(50 milliseconds)

END LOOP
